# Molecular Characterization of Native Entomopathogenic Fungi from Ambrosia Beetles in Hazelnut Orchards of Turkey and Evaluation of Their In Vitro Efficacy

**DOI:** 10.3390/insects13090824

**Published:** 2022-09-11

**Authors:** Rahman Kushiyev, Celal Tunçer, İsmail Oğuz Özdemir, İsmail Erper, Ruslan Kalendar, Mehtap Alkan, Göksel Özer

**Affiliations:** 1 Department of Plant Protection, Faculty of Agriculture, Ondokuz Mayis University, 55139 Samsun, Turkey; 2 Department of Plant Protection, Faculty of Agriculture, Sakarya University of Applied Sciences, 54580 Sakarya, Turkey; 3 Department of Plant Protection, Faculty of Agriculture, Kyrgyz Turkish Manas University, Bishkek 720044, Kyrgyzstan; 4 Institute of Biotechnology, HiLIFE, University of Helsinki, Biocentre 3, 00790 Helsinki, Finland; 5 Department of Plant Protection, Faculty of Agriculture, Bolu Abant Izzet Baysal University, 14030 Bolu, Turkey

**Keywords:** *Anisandrus dispar*, *Xylosandrus germanus*, *Xyleborinus saxesenii*, biocontrol, molecular characterization, iPBS profiling, insect-pathogenic fungi

## Abstract

**Simple Summary:**

Turkey is the world’s largest producer and exporter of hazelnut. Ambrosia beetle species are the most common species of pests for hazelnut in the orchards of Turkey. These beetles cause enormous economic losses by draining hazelnut branches and trees. The techniques for managing ambrosia beetles are limited. The more effective and eco-friendly alternative control methods, including the use of entomopathogenic fungi (EPF), should be included in integrated pest management programs to suppress ambrosia beetle populations. The objectives of the current study were (i) to isolate EPF from individual ambrosia beetles that were obtained from Turkey’s main hazelnut production areas; (ii) to characterize EPF isolates using DNA sequencing and iPBS profiling; and (iii) to assess the effectiveness of the isolates against three ambrosia beetle species under laboratory conditions. A total of 47 EPF isolates were obtained from beetle cadavers and classified into eight EPF species. For the first time, the primer binding site (PBS) marker system was used to successfully discriminate among the EPF species. Some isolates caused 100% mortality of the beetle species within 7 to 9 days, depending on the beetle species, demonstrating their effectiveness in managing the pests. The major EPF species in this study provided an important basis for developing bioproducts and a possible alternative approach in controlling these ambrosia beetles.

**Abstract:**

Ambrosia beetles, *Anisandrus dispar* Fabricius, *Xylosandrus germanus* Blandford, and *Xyleborinus saxesenii* Ratzeburg (Coleoptera: Curculionidae: Scolytinae) are among the most significant hazelnut pests in Turkey. The control of these pests is difficult and expensive due to their biology. The present study aimed to isolate entomopathogenic fungi (EPF) from *A. dispar*, *X. germanus*, and *X. saxesenii* individuals that were obtained from the main hazelnut production areas of Turkey, characterize the EPF isolates using internal transcribed spacer (ITS)-DNA sequencing and iPBS profiling, and determine the efficacy of the isolates against *A. dispar*, *X. germanus*, and *X. saxesenii* under laboratory conditions. Phylogenetic analyses based on ITS revealed that the 47 native isolates were *Beauveria bassiana* (11), *B. pseudobassiana* (8), *Cordyceps fumosorosea* (6), *Cordyceps farinosa* (1), *Akanthomyces lecanii* (13), *Purpureocillium lilacinum* (3), *Clonostachys rosea* (2) and *Metarhizium anisopliae* (3). For the first time, the primer binding site (PBS) marker system, based on retrotransposons, was used to discriminate successfully among the EPF species. Some isolates of *B. bassiana*, *B. pseudobassiana*, *C. fumosorosea*, *A. lecanii*, and *M. anisopliae* caused 100% mortality of the beetle species within 7 to 9 days. The findings of this study indicated that some isolated entomopathogenic fungi provide an essential basis for the development of bioproducts, as well as a promising alternative method for controlling these ambrosia beetles.

## 1. Introduction

Turkey is the world’s largest producer and exporter of hazelnut, with 512.000 metric tonnes of hazelnut production (with shell), which equals 62% of the global output [1]. The average yield in Turkey is approximately 905 kg/ha, which is 1.93 and 2.93 times less, respectively, than the average yields in Italy and the United States of America [2]. Diseases and pests that threaten production are among the main causes of low yield [3].

Among 3400 species of ambrosia beetles, bark and ambrosia beetles (Coleoptera: Curculionidae: Platypodinae and Scolytinae) are significant pests for many fruits and forest trees, as well as for hazelnut [4,5]. Ambrosia beetles are wood-boring insects that bore tunnels through the sapwood (xylem) of trees. In these galleries, the beetles cultivate symbiotic fungi, such as *Ambrosiella* spp. And *Raffaelea* spp., to supply sustenance for larvae and adults [6,7]. Furthermore, following their penetration into trees, ambrosia beetles commonly inoculate harmful secondary fungi [8], such as *Fusarium* spp. [9] or bacteria [10]. These beetles also harm plants by carrying disease by cultivating symbiotic fungi in the tunnels they excavate in trees [7].

The ambrosia beetle species *Anisandrus dispar* Fabricius, *Xylosandrus germanus* Blandford, and *Xyleborinus saxesenii* Ratzeburg are the most common species of pests for hazelnut in the orchards of Turkey [5]. These beetles cause enormous economic losses by draining hazelnut branches and trees, particularly in the orchards along the Black Sea coast of the country, which has high groundwater levels, and in the orchards that are located near woodlands [3]. In managing ambrosia beetles, chemical, cultural, and biotechnical control practices against adults are used to a limited extent [11]. However, they cannot satisfactorily prevent the spread of these beetles to new orchards or the harm caused by them. Insecticide application is needed at least 5 or 6 times against ambrosia beetles in hazelnut orchards [11]. Chemical control methods are ineffective against these pests, due to the high cost of such methods, their low efficacy, and their detrimental environmental effects. The more effective and eco-friendly alternative control methods, including entomopathogenic fungi (EPF), should be included in integrated pest management methods to suppress ambrosia beetle populations [11,12].

*Beauveria bassiana*, *Metarhizium anisopliae*, *Cordyceps fumosorosea* (formerly: *Isaria fumosorosea* and *Paecilomyces fumosoroseus*), and *Lecanicillium* (Akanthomyces) spp. infect insects by penetrating their cuticles to feed on their bodies, eventually killing them [13,14,15]. Ambrosia beetles that live in the galleries of wood tissue are not ideal targets for insecticides, but applications of EPFs may be more effective. A large number of studies have shown the effectiveness of EPFs, including *B. bassiana*, *M. anisopliae*, *C. fumosorosea* against *A. dispar*, *X. germanus*, *Xylosandrus crassiusculus* Motschulsky, *Xyloborus glabratus* Eichhoff, and *Trypodendron lineatum* Oliv. (Col.: Scolytidae) [11,16,17,18,19,20,21]. Tuncer et al. [11] reported that applications of 1 × 10^8^ conidia mL^−1^ concentrations of *M. anisopliae* on the bodies of insects and branches, against the adult females of *X. germanus*, caused 100% mortality, while the application of *B. bassiana* on insects and branches caused 80% and 64% mortality, respectively. In addition, EPFs not only caused the death of adults, but they were also carried to galleries by adults, resulting in infections of other individuals and eggs in the gallery [16,17,18,19]. 

In particular, native EPF isolates that are environmentally compatible with pest species reduce the risk of significant impacts on non-target organisms, compared with exotic isolates [22]. Studies on naturally occurring EPFs that are associated with these beetles may identify strains that could be more virulent against the beetles, or more effectively obtained from natural habitats [23]. 

There is currently no effective control strategy for controlling ambrosia beetles in hazelnut orchards. The Black Sea region of Turkey, the world’s largest hazelnut growing area, has ideal climate conditions for the growth of entomopathogenic fungus, including rain, humidity, and a low average temperature [24]. EPFs have a high potential as an alternative control method against ambrosia beetles, which spend almost all of their lives in tunnels in different developmental stages. Studies on the evaluation and isolation of entomopathogenic fungi in controlling ambrosia beetles in hazelnut orchards are very limited. Therefore, obtaining, identifying, and testing the efficacy of naturally occurring EPF in controlling ambrosia beetles is a critical step toward developing an alternative control strategy.

On the other hand, the characterization of EPF isolates is critical in understanding the natural biodiversity of fungi in a given region and provides a pool of potential biological control agents for pest control purposes [25]. DNA sequencing and DNA marker systems have been frequently used to characterize fungal isolates at inter- or intra-species levels. Internal transcribed spacer (ITS)-DNA sequencing is used to discriminate among species of EPF [26]. Inter-primer binding site (iPBS) profiling markers, based on repeat sequences for retrotransposons containing the tRNA priming binding site, are excellent and sensitive tools for detecting genetic variations in eukaryotic genomes [27]. The marker system has been used to evaluate fungal genomes [28,29], but not previously for EPF species.

The aims of the present study were (i) to isolate EPF from *A. dispar*, *X. germanus*, and *X. saxesenii* individuals obtained from the main hazelnut production areas of Turkey, (ii) to characterize EPF isolates using ITS sequencing and iPBS profiling, and (iii) to determine the efficacy of the isolates against *A. dispar*, *X. germanus*, and *X. saxesenii* under laboratory conditions. Thus, we carried out one of the crucial steps in the alternative microbial control of ambrosia beetles in hazelnut orchards.

## 2. Materials and Methods

### 2.1. Beetles Collection

Hazelnut orchards in different districts of the Samsun, Ordu, Giresun, Düzce, and Sakarya provinces were surveyed to collect hazelnut branches that were infested with *A. dispar*, *X. germanus*, and *X. saxesenii*. The infested branches (0.5 m- and 1m-long pieces) were directly transferred to the Entomology Laboratory, Ondokuz Mayis University, Samsun, Turkey. The branches were dissected by pruning scissors and hammers on a white sheet, and the beetles in the galleries were collected with brushes. They were inspected under an EZ4 stereomicroscope (Leica Microsystems, Wetzlar, Germany) and classified according to species [5] and live/dead/infected status [19]. The individuals were stored at 4 °C.

### 2.2. Isolation of Entomopathogenic Fungi from Ambrosia Beetles

In isolating EPF, cadavers and infected individuals of ambrosia beetles were used. These beetles were surface-disinfected with 1% NaOCl (sodium hypochlorite) for 3 min, rinsed with sterile distilled water three times, and placed on sterile blotting paper to dry for 30 min. The insect cadavers were placed into 9 cm Petri dishes containing potato dextrose agar (PDA; Merck Ltd., Darmstadt, Germany), in two- or four-beetle sets. All Petri dishes were maintained in a KBWF 240 incubator (BINDER GmbH Tuttlingen, Germany) at 25 °C in the dark. After incubation, a small piece of mycelium from the fungi colonies growing on the medium with different colony morphologies was taken to prepare a spore suspension at the rate of 1 × 10^4^ spore mL^–1^. A 20 µL aliquot of the spore suspension was spread on a PDA plate and incubated at 25 °C in the dark. The plates were examined with a dissecting microscope, and a single germinating spore was selected from each plate with a sterile surgical blade and transferred to a new plate containing PDA. Sterile pieces (1 cm^2^) of Whatman’s filter paper were placed on the media and incubated at 25 °C for 1 to 2 weeks. At the end of incubation, the pieces of filter paper covered with fungal mycelia/spores were placed into sterile Eppendorf tubes and stored at −20 °C [26].

### 2.3. DNA Extraction of Fungal Isolates

The fungal isolates of mycelia and spores listed in Table 1 were scraped from the surface of the 10 day PDA cultures with a sterile scalpel and transferred into 1.5 mL Eppendorf tubes. According to the manufacturer’s guidelines, DNA extraction was performed by employing DNeasy Blood and Tissue kit (Qiagen, Cat No./ID; 69504, Hilden, Germany). The resultant DNA was diluted to 20 ng/μL with sterile ultra-pure water and measured with a DS-11 FX+ spectrophotometer (Denovix Inc., Wilmington, DE, USA). The DNA templates were stored at −20 °C till used.

### 2.4. ITS Sequencing and Phylogenetic Analysis

The internal transcribed spacer (ITS) fragment of ribosomal DNA was amplified using ITS1/ITS4 [30]. The PCR mixture contained a 1× PCR reaction buffer, 0.2 mM of each dNTP, 0.4 µM of each primer, 1.5 U Ampliqon TEMPase Hot Start DNA polymerase (Berntsen, Rdovre, Denmark), 10 ng template DNA, and Milli-Q water up to 50 μL. The PCR amplification procedures were as follows: 95 °C for 15 min; 95 °C for 60 s, annealing at 54 °C for 60 s, extension at 72 °C for 60 s for 35 cycles; and a final extension at 72 °C for 10 min, conducted in a T100 thermal cycler (Bio-Rad Laboratories, Hercules, CA, USA). The PCR products were sent to the Macrogen Inc. sequencing service (Seoul, Korea) for bidirectional sequencing with the same primers.

The DNA sequences were edited, and consensus sequences were estimated manually using MegaX software [31]. All of the sequences were compared against the GenBank nucleotide database (https://www.ncbi.nlm.nih.gov/genbank/, accessed on 1 July 2022) using the BLASTn algorithm. All of the generated sequences of the EPF isolates were deposited in GenBank. GenBank provided the accession numbers of the submitted sequences, as shown in Table 1. The phylogenetic relationships of all of the newly generated sequences of the EPF isolates that were obtained in this study, the reference sequences retrieved from GenBank, and *Penicillium chrysogenum* (CBS 129601) as a monophyletic outgroup were investigated, on the basis of ITS sequences. DNA sequences were aligned, using the Clustal W program [32] and the MEGA7 software package [33]. The phylogenetic tree was inferred, using the maximum likelihood method based on the Tamura-Nei model, which was applied to the whole set of the aligned sequences. The confidence of the branching was calculated by bootstrap analyses (1000 replicates).

### 2.5. iPBS Profiling Analysis

A total of 10 PBS primers designed by Kalendar et al. [27] were employed for screening inter- or intra-species genetic variation, based on DNA profiling methods by PCR amplification (Table 2). The iPBS-amplification PCR was conducted in a 25 µL reaction volume that included 25 ng of genomic DNA, 1x Ampliqon *Taq* DNA Polymerase Master Mix (Berntsen, Rdovre, Denmark), and 1 µM PBS primer. The PCR program consisted of a pre-denaturation of 3 min at 95 °C, followed by 35 cycles of 30 s at 95 °C, 30 s at 50–62 °C (depending on the PBS primer), 1 min at 72 °C, and, finally, an extension cycle of 5 min at 72 °C. The PCR products were separated in 1.4% agarose gel using the 1x TAE buffer at 70 V for 2 h and stained with ethidium bromide for visualization under ultraviolet (UV-B) light in the G: BOX F3 gel documentation system (Syngene, Synoptics Ltd., Cambridge, UK).

PCR amplification of the iPBS markers was repeated twice, and only reproducible and clear bands were recorded to construct a “0:1” binary matrix. The polymorphism information content (PIC) and resolving power (RP) were estimated by using the formula of Roldán-Ruiz et al. [34] and Prevost and Wilkinson [35], respectively. We examined the genetic variation both between and within the defined species using GenAlEx 6.5 [36]. The number of polymorphic bands (PPB) and the percentage of polymorphic bands (PPB) were calculated to estimate genetic diversity between and within fungal species. For each species, the parameters included the observed and effective number of alleles, Nei’s gene diversity (h), and Shannon’s information index (I). The coefficient for the genetic distance between the fungal pathogens was estimated according to Jaccard’s index for pairwise comparisons, based on the proportion of shared alleles for all primers. Unweighted pair group method analysis (UPGMA) and principal coordinate analysis (PCoA) were performed with the vegan library Ver. Vegan 2.4.4 in R [37].

### 2.6. Collection of Healthy Beetles

Branch samples infested with beetles were obtained from hazelnut orchards in the Ondokuz Mayıs and Çarşamba districts in Samsun province. The branches were then brought to the laboratory. Female adults of *A. dispar*, *X. germanus*, and *X. saxesenii* were obtained using the method mentioned above and used in all bioassay studies. The obtained beetles were inspected under a Leica EZ4 stereomicroscope at ×60 magnification to separate live and healthy female adults. The adults were of standard size and symptom-free.

### 2.7. Bioassays

All isolates obtained from ambrosia beetles were cultured on PDA media for 2 to 3 weeks in the dark at 25 °C to ensure sporulation. Spores were obtained by scraping with glass drumsticks and 10 mL of sterile distilled water containing 0.1% Tween 80. The spore suspensions were filtered into 50 mL sterile glass flasks with double-layered cheesecloth to remove the mycelial and agar pieces and homogenized by vortexing for 5 min. Spore concentrations were counted with a hemocytometer and adjusted to the desired 1 × 10^8^ mL^−1^ conidial concentration, which was used throughout the entire bioassay studies to provide an overall projection of efficacy for all isolates. To test the viability of the spores of each isolate, 100 µL of spore suspension from a concentration of 1 × 10^4^ spore mL^−1^ was spread in 6 cm Petri dishes containing the PDA medium and incubated at 25 °C. After 24 h of incubation, 200 spores from each Petri dish were examined, and the viability rate was determined [26]. Spores with germ tubes larger than the spore diameter were considered germinated, and suspensions with more than 95% germinating spores were used in pathogenicity tests [21].

Surface disinfection of female beetles used in the bioassays was performed with 70% ethanol for 10 s, and these beetles were placed on autoclaved filter papers for drying [19]. The hazelnut branches (4 cm in length and 1.5 cm in diameter) used in the bioassays were sterilised by autoclaving at 121 °C for 30 min on two consecutive days. The bottom of the Petri dishes used in all experiments was covered with moistened sterile blotting papers. Later, 2 mL was taken from of 1 × 10^8^ spore mL^−1^ suspension obtained from all isolates and directly sprayed on five females of *A. dispar*, *X. germanus*, and *X. saxesenii* placed in each Petri dish by using a Potter spray tower (Burkard Manufacturing Co., Ltd., Rickmansworth, UK), and the sterilized branch was placed in each Petri dish. After each application, the spraying tower was disinfected with 70% ethyl alcohol and washed with sterile distilled water. Sterile distilled water containing only 0.01% Tween 80 was sprayed in the control Petri dishes with the same method, and a sterile hazelnut branch was placed in all Petri dishes. The edges of all Petri dishes were then covered by Parafilm and incubated at 25 ± 1 °C, 70 ± 5% RH, and 16:8 h light/dark for 9 days in the KBWF 240 incubator. Each treatment in the trials had five replications with five female beetles. Mortalities were recorded for 9 successive days to ensure the independence of each day’s observations from each other. The trial was replicated by using the same number of different individuals (*n* = 25 insects/day/isolate/1 × 10^8^ spore mL^−1^ concentration) for each day, and after determining the mortalities of the beetles of the relevant day on each counting day, the beetles and Petri dishes belonging to that day were removed from the trial [38]. The same procedure was repeated for the control groups.

### 2.8. Statistical Analyses

Because the mortality rates in the pathogenicity testing exceeded 5%, these results were corrected using Abbott’s formula [39]. The log-probit algorithm with the probit analysis program (POLO-PLUS ver. 2.0) was used to determine independent-time mortality data statistics, reported as 50% lethal time (LT_50_) and 90% lethal time (LT_90_) from the bioassays. Standard errors were used to compare the slopes of the regression lines, and the isolates’ LT_50_ and LT_90_ values were compared using confidence ranges (95%). All statistical data associated with the parameters are provided as Tables and Figures in the Supplementary Materials.

## 3. Results

### 3.1. Identification of Isolates

**Figure 1:**
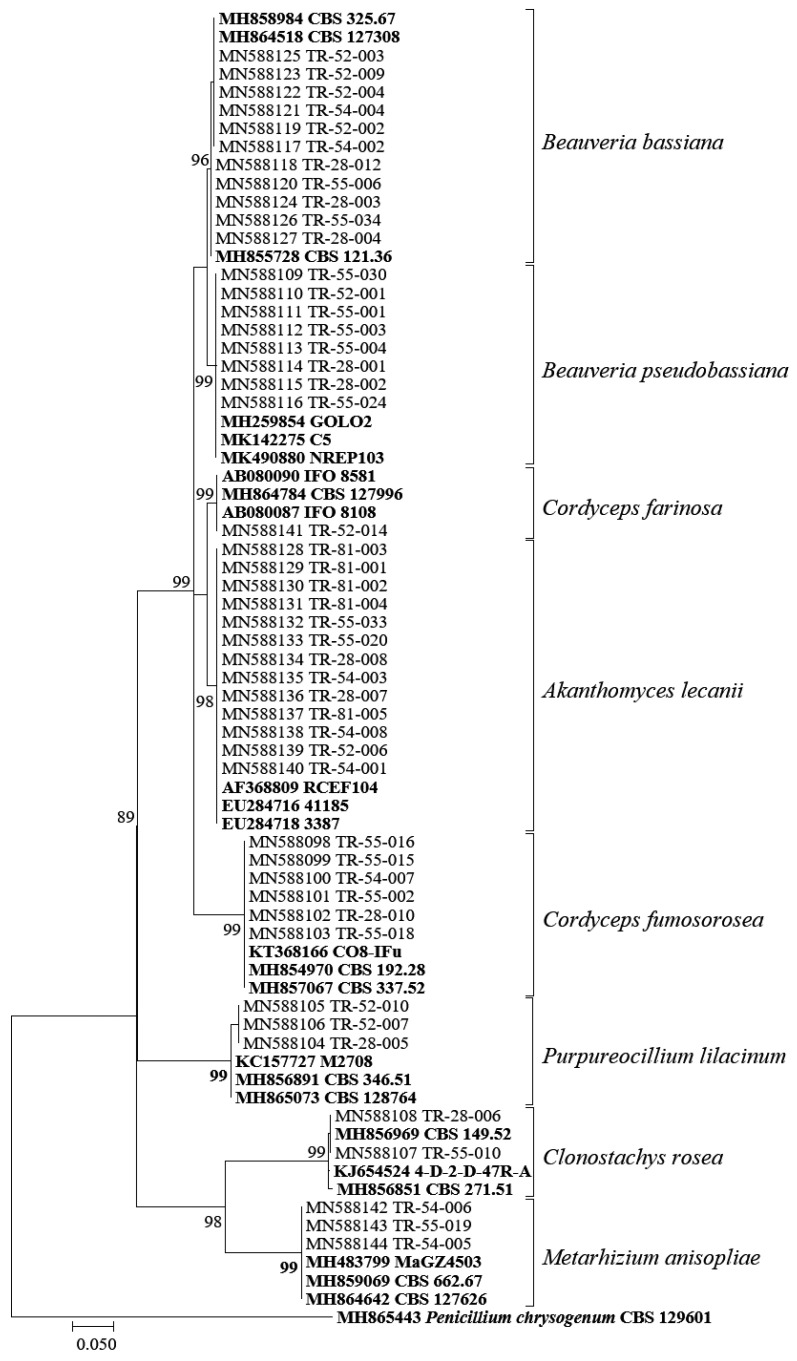
Maximum-likelihood (ML) phylogenetic tree generated from the ITS dataset of entomopathogenic fungal isolates. Bootstrap values are shown for each node.

A total of 47 EPF isolates were obtained from cadavers of *A. dispar*, *X. germanus*, and *X. saxesenii* collected from the main hazelnut-producing provinces in Turkey. It was determined that 23 isolates were obtained from *A. dispar*, 19 isolates from *X. germanus*, and five isolates from *X. saxesenii* (Table 1). The total length of the internal transcribed spacer fragments deposited in GenBank ranged from 511 bp to 550 bp. The molecular characterization of the isolates was verified using the BLASTn algorithm running on the NCBI website for each species, and the ITS isolates showed 99% to 100% nucleotide identity with those of the corresponding isolates derived from Genbank. The isolates were classified into eight EPF species, as shown in Table 1. A maximum-likelihood phylogenetic analysis based on the ITS data confirmed the clustering of the isolates with the reference isolates (Figure 1).

### 3.2. iPBS Profiling Analysis

**Figure 2:**
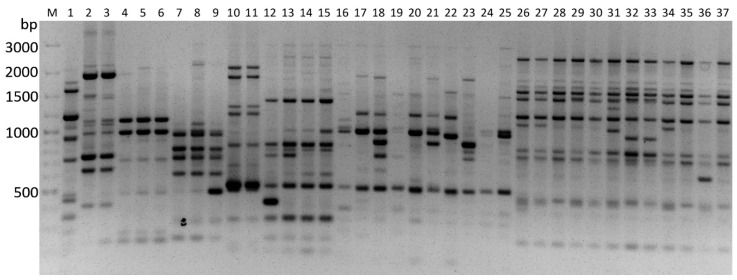
The band profiles with PBS primer (2395) for entomopathogenic fungal isolates. M: GeneRuler 100 bp Plus DNA Ladder (Thermo Fisher Scientific, Waltham, MA, USA). 1: *Cordyceps farinosa*, 2–3: *Metarhizium anisopliae*, 4–6: *Cordyceps fumosorosea*, 7–9: *Purpureocillium lilacinum*, 12–15: *Beauveria pseudobassiana*, 16–25: *Beauveria bassiana* 26–37: *Akanthomyces lecanii*.

The 10 PBS primers specified in Table 2 were found to be reproducible and were able to differentiate all of the EPF species efficiently. The amplification profiling of the primers yielded 237 reproducible and scorable fragments to evaluate the genetic variation among the isolates. The representative pattern of iPBS profiling of eight EPF species from cadavers of *A. dispar*, *X. germanus*, and *X. saxesenii* using 10 PBS primers is demonstrated in Figure 2. The mean percentage of the polymorphic band for the primers was 94.87. The number of bands produced with the PBS primers ranged from 18 (2080/2381) to 30 (2390), with a mean of 23.7 bands per primer.

The mean PIC and RP values calculated for the primers are provided in Table 2. These parameters represent the characteristic features of a marker used and provide information regarding the discriminating power. The range of the PIC was 0.20 (2395) to 0.26 (2221), averaging 0.24. The mean of the RP values, a parameter that signifies the discriminatory potential for the selected primers, was 7.14 for all primers. The lowest RP value of 4.77 was obtained from 2381, while the highest RP value was recorded as 9.06 for 2221.

**Figure 3:**
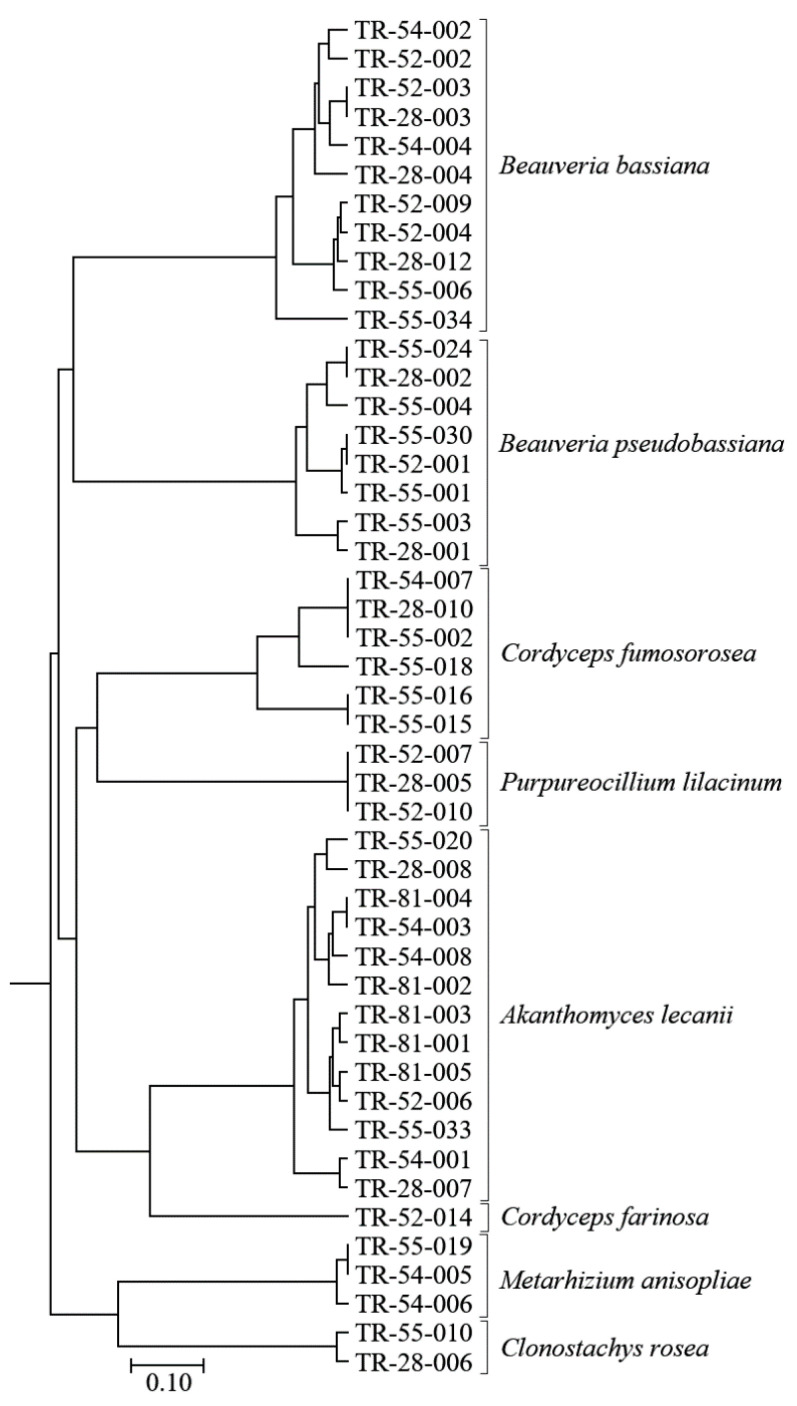
UPGMA cluster analysis based on Jaccard similarity coefficients, showing the genetic relationships among eight EPF species based on an iPBS profiling analysis.

An unweighted pair group method analysis, based on Jaccard similarity coefficients, revealed the genetic relationships among the 47 isolates belonging to the eight EPF species. Isolates were grouped into eight main clusters, which were entirely conserved among isolates within the same species allocation (Figure 3). This clustering, based on iPBS-amplification profiling data, confirmed the grouping of isolates based on ITS sequences. The isolates in the same species group were further differentiated from the others by different similarity degrees. *Clonotachys rosea* and *M. anisopliae* isolates formed a distinctly different group from other isolates, supporting the grouping ML phylogenetic tree that was based on the ITS dataset.

All ten PBS primers provided a great degree of discrimination among the EPF species. The parameters associated with genetic variations among the species using GenAlex were as follows: the number of alleles equalled 5.875 ± 0.095; the number of effective alleles equalled 1.048 ± 0.004; and the Shannon’s information index equalled 0.045 ± 0.003. The Nei’s genetic distance index between all species pairs was calculated by GenAlex (Table 3). Accordingly, the genetic distance values varied between 0.072 and 0.369, with an average of 0.207. The lowest distance was observed between *C. farinosa* and *A. lecanii* species, and the highest distance4 was between *M. anisopliae* and *B. pseudobassiana*. Unfortunately, the sample size for some species was less than five.

### 3.3. Bioassay Studies

#### The Efficacy of the Entomopathogenic Fungi against Ambrosia Beetles

A total of 11 *B. bassiana* isolates were applied to *A. dispar* at a concentration of 1 × 10^8^ spore mL^−1^, and the lowest LT_50_ (4.12) and LT_90_ (5.96) values (days) (*y* = −4.9 + 7.97*x*; *x^2^* = 40.6; *df* = 43) were observed in the TR-55-006 isolate. This isolate was statistically different from the others (*p* < 0.05). The TR-55-006 and TR-55-034 isolates caused 100% mortality by day 7, and the other isolates caused 30% to 100% mortality by day 9. Similarly, these isolates were used against *X. germanus* at the same concentration, and the lowest LT_50_ (3.97) and LT_90_ (5.67) values (days) (*y* = −4.9 + 8.34*x*; *x*^2^ = 41.37; *df* = 43) were recorded for the TR-55-006 isolate (*p* > 0.05). The mortality rate caused by the TR-55-006 and TR-55-034 isolates was 100% on the seventh day, while the mortality rates for the other isolates ranged between 30% and 100% on the ninth day.

It was determined that the most effective isolate within the eight *B. pseudobassiana* isolates applied against *A. dispar* was TR-52-001, with LT_50_ (4.56) and LT_90_ (6.28) values (days) (*y* = −6.09 + 9.25*x*; *x*^2^ = 13.02; *df* = 43; *p* > 0.05), which caused 100% mortality 7 days after the treatment. The other isolates caused 10% to 100% mortality 9 days after the treatment. The same isolates were applied to *X. germanus*, and the lowest LT_50_ and LT_90_ values were 5.16 and 8.50 days (*y* = −4.21 + 5.92*x*; *x*^2^ = 36.73; *df* = 43) for TR-55-001, 5.35 and 8.07 days (*y* = −5.22 + 7.18*x*; *x*^2^ = 15.55; *df* = 43) for TR-52-001 and 5.40 and 7.86 days (*y* = −5.74 + 7.84*x*; *x*^2^ = 12.78; *df* = 43) for TR-55-030 (*p* > 0.05). The tree isolates caused 100% mortality 9 days after treatment, while the other isolates caused 15% to 100% death 9 days after application.

As a result of the application of three isolates of *M. anisopliae* against *A. dispar*, the LT_50_ (3.63) and LT_90_ (5.02) values for the TR-55-019 isolate (*y* = −5.1 + 9.1*x*; *x*^2^ = 25.94; *df* = 43) were found to be lower than those of other isolates, and the isolate statistically differed from the others in terms of LT_50_ value (*p* < 0.05). The TR-55-019 isolate caused 100% mortality by day 7, while the other isolates caused 100% mortality by days 7 (TR-54-005) and 9 (TR-54-006). Similarly, the LT_50_ and LT_90_ values of the TR-55-019 isolate applied to *X. germanus* were determined as 4.01 days and 5.40 days (*y* = −5.96 + 9.89*x*; *x*^2^ = 6.73; *df* = 43), respectively. The LT_90_ value of these isolates was statistically different from the others (*p* < 0.05). The TR-55-019 isolate caused 100% mortality 7 days after treatment, while the other isolates caused 100% mortality 9 days after treatment.

Seven *C. fumosorosea* and *C. farinose* isolates were applied to *A. dispar* to evaluate their efficacy. Several isolates (TR-55-002, TR-55-018) were more virulent than the other isolates. The lowest LT_50_ and LT_90_ values (days) were recorded for the TR-55-002 isolate [LT_50_ (5.19) and LT_90_ (8.21) (*y* = −4.62 + 6.45*x*; *x*^2^ = 19.82; *df* = 43)] and the TR-55-018 isolate [LT_50_ (5.12) and LT_90_ (7.84) (*y* = −4.92 + 6.94*x*; *x*^2^ = 13.6; *df* = 43)], and these isolates were statistically different from the other isolates (*p* < 0.05). Both isolates caused a 100% mortality rate by day 9, while the mortality rates of the others ranged between 30% and 52%, depending on the isolates, by day 9. The lowest LT_50_ and LT_90_ values (days) in applying the same isolates to *X. germanus* were determined to be in the TR-55-002 isolate [LT_50_ (5.36) and LT_90_ (8.33) (*y* = −4.87 + 6.68*x*; *x*^2^ = 14.43; *df* = 43)], and this isolate statistically differed from the other isolates (*p* < 0.05). The TR-55-002 isolate caused 100% mortality at the end of 9 days, while the others caused 30% to 90% mortality 9 days after application.

The LT_50_ and LT_90_ values of the TR-81-004, TR-54-003, TR-28-008, and TR-28-007 isolates were lower, due to the application of 13 different isolates of *A. lecanii* against *A. dispar*, and they were statistically different from the others (*p* < 0.05). The mortality rates belonging to these isolates against *A. dispar* were 100%, while these rates for the others attained 20% to 60% by day 9. Among these isolates, the lowest LT_50_ and LT_90_ values were found at 4.83 days and 7.05 days (*y* = −5.34 + 7.81*x*; *x*^2^ = 12.9; *df* = 43; *p* < 0.05) for TR-54-003. Similarly, the efficacy of the same isolates of *A. lecanii* applied to *X. germanus* was evaluated, and the lowest LT_50_ and LT_90_ values were found as 4.43 days and 6.06 days (*y* = −6.09 + 9.43*x*; *x^2^* = 10.59; *df* = 43; *p* < 0.05) for the TR-81-004 isolate, which was statistically different from the others (*p* < 0.05). The TR-81-004 isolate caused 100% mortality 7 days after treatment, while the others caused 10% to 100% mortality 9 days after treatment.

Three isolates of *P. lilacinum* applied to *A. dispar* were found to have insignificant differences (*p* > 0.05), and the lowest LT_50_ (6.68) value [*y* = −4.69 + 5.69*x*; *x^2^* = 10.42; *df* = 43] for the TR-28-005 isolate and the LT_90_ (10.69) value [*y* = −5.42 + 6.52*x*; *x*^2^ = 11.07; *df* = 43] for the TR-52-007 isolate occurred. Both isolates caused 72% mortality on the ninth day of the application, while the other caused 64% mortality on the ninth day of the application. Similarly, the same isolates applied to *X. germanus* were statistically found to be similar to each other (*p* > 0.05). The TR-28-005 isolate had the lowest LT_50_ (7.19 days) and LT_90_ (12.63 days) values (*y* = −4.49 + 5.24*x*; *x*^2^ = 10.02; *df* = 43; *p* > 0.05) and caused 68% mortality by day 9. The mortality rates in the other isolates ranged between 64% and 68% at the end of 9 days.

The efficacy of two *C. rosea* isolates obtained in the study was evaluated, and the LT_50_ and LT_90_ values for the TR-55-010 isolate were 11.66 and 19.43 days (*y* = −6.15 + 5.77*x*; *x*^2^ = 11.14; *df* = 43; *p* > 0.05), respectively. The TR-55-010 isolate caused 20% mortality at the end of 9 days. The LT values could not be calculated because the TR-28-006 isolates did not cause mortality until the seventh day of the application. Similarly, the two isolates applied to *X. germanus* caused 10% to 20% mortality by day 9. The LT values for these isolates could not be determined, due to the very low mortality rates.

Thirteen isolates of *B. bassiana*, *B. pseudobassiana*, *C. fumosorosea*, *A. lecanii*, and *M. anisopliae* species, which showed significant efficiency against two other beetles, were chosen and applied to *X. saxesenii*, which had the lowest occurrence in the surveys in hazelnut orchards. The lowest LT values were recorded as LT_50_ (3.71) and LT_90_ (5.57) values (days) (*y* = −4.13 + 7.26*x*; *x^2^* = 21.29; *df* = 43) for the TR-55-006 (*B. bassiana*) isolate and LT_50_ (4.28) and LT_90_ (6.12) values (*y* = −5.20 + 8.23*x*; *x^2^* = 11.41; *df* = 43) for the TR-55-019 (*M. anisopliae*) isolate (*p* > 0.05). The TR-55-034 and TR-54-005 isolates, along with the *B. bassiana* and *M. anisopliae* isolates, caused 100% mortality by day 7, while the other isolates caused 30% to 100% mortality by day 9.

## 4. Discussion

A total of 47 EPF isolates were obtained from *A. dispar*, *X. germanus*, and *X. saxesenii*. *A. lecanii* was found to be the most common entomopathogenic fungus species among all of the isolated fungi. *Beauveria pseudobassiana*, *C. fumosorosea*, *C. farinosa*, *A. lecanii*, *P. lilacinum*, and *C. rosea* were first detected as entomopathogenic fungi from these beetles, based on the identification and isolation of entomopathogenic fungi obtained from *A. dispar*, *X. germanus*, and *X. saxesenii* in Turkey and worldwide. The sequence analysis of the ITS region provided for the discrimination and identification of EPF isolates, according to their species. Özer et al. [28] showed the efficacy of the iPBS molecular marker system in the characterization of different fungal isolates. Thereafter, iPBS profiles were successfully used to characterize various fungi and yeasts [27,28,29,40,41]. To the best of our knowledge, this study is the first to use the iPBS marker system for the DNA fingerprinting of isolates that belong to eight EPF species. Following the previous studies carried out with PBS primers to characterize fungal isolates, we concluded that the PIC and RP values of the PBS primers in this study were quite satisfactory. Their use alone or in combination will allow them to be applied in the differentiation of EPF species.

In the previous studies, *L. muscarium* was isolated from *A. dispar* and *X. germanus* in Turkish hazelnut orchards [42] and *Dendroctonus micans* Kug. (Col.: Scolytidae), which is the most common bark beetle in the forests of Turkey [43]. Two different isolates of *L. muscarium* caused 35% to 40% mortality on *D. micans* individuals 10 days after the treatment [43], while in the present study, the TR-81-004 isolate of *A. lecanii* showed the highest virulence against *A. dispar*, *X. germanus*, and *X. saxesenii* by causing 100% mortality 9 days after treatment.

The *Beauveria* genus that was isolated in this study is known to be one of the most common EPFs globally, with a wide range of hosts worldwide, including some bark and ambrosia beetles [13,43,44]. There are many commercial bio-insecticides that are widely used against agricultural pests, which are produced from this fungus [13]. This fungus was previously isolated from *X. germanus* and *Hyphantria cunea* Drury (Lepidoptera: Arctiidae) [42], from *Curculio nucum* L. (Coleoptera: Curculionidae) [45], and from soils [22]. In our pathogenicity assays, the TR-55-006 from all *B. bassiana* isolates and some *B. pseudobassiana* isolates were used against *A. dispar*, *X. germanus*, and *X. saxesenii*. The isolate showed significant efficacy (100% mortality) by days 7 and 9, respectively. In parallel with the results of the present study, it was reported that *B. bassiana* isolates were highly virulent against some bark (*Scolytus amygdali* Geurin-Meneville (Col.: Scolytidae), *D. micans*, *Ips sexdentatus* Boerner (Col.: Scolytidae), and *Ips typographus* L. (Col.: Scolytidae)) [43,46,47,48,49] and ambrosia beetles (*T. lineatum*, *A. dispar*, and *X. germanus*) [11,16,17,18,19].The isolates had considerable potential in terms of controlling the beetles. In a similar study, the LT_50_ and LT_90_ values of the *B. bassiana* (TR-217) isolate applied directly on *X. germanus* at a concentration of 1 × 10^8^ spore mL^−1^ were 5.96 and 11.79 days, respectively, and the isolate caused 80% mortality by day 8 [11]. Among the promising biocontrol agents against ambrosia beetles, *B. bassiana* Naturalis^®^ was sprayed on *X. germanus* at a concentration of 600 conidia/mm^2^ under laboratory conditions, with a high survival efficiency of >60% mortality after 6 days post-treatment [18]. In addition, *B. pseudobassiana* was found to be highly effective against bark beetles, such as *D. micans*, *I. sexdentatus*, and *I. typographus* [50,51]. The present study showed that *B. bassiana* and *B. pseudobassiana* had high pathogenicity against *A. dispar*, *X. germanus*, and *X. saxesenii*. Therefore, it is thought that these native isolates, showing high virulence, could be potential biocontrol agents in the management of the beetles.

*Cordyceps fumosorosea* and *I. farinosa*, isolated in the present study, were previously isolated from soils taken from hazelnut orchards in the Black Sea region [22] and bark beetles, such as *D. micans*, *I. typographus*, and *T. lineatum* [43,44,52]. In the pathogenicity study, TR-55-002 of *C. fumosorosea* was the most effective isolate for each beetle and caused 100% mortality within 9 days. Although the *Isaria* genus is used to control many pests worldwide [15], studies on the ambrosia beetles are very limited. In a study conducted by Kushiyev et al. [21], the LT_50_—LT_90_ values of the *C. fumosorosea* (TR-78-3) isolate on *A. dispar* and *X. germanus* adults were recorded, respectively, as 4.78 days to 5.94 days and 4.18 days to 5.62 days at a concentration of 1 × 10^8^ spore mL^−1^, and a 100% mortality rate for the beetles occurred at the end of day 8 [21].

*Metarhizium anisopliae* isolated from ambrosia beetles in the present study is the most studied EPF as a biological control agent against many agricultural pests [14,18,19]. It has been determined that this fungus is a potential EPF against many important pests, and it is also successfully used as a commercial bio-product [14]. This fungus has previously been isolated from *X. germanus* [53], and there is no record of its being isolated from other ambrosia beetles. *Metarhizium anisopliae* has also been intensely isolated from the soils taken from hazelnut orchards in the Black Sea region, and it could be a promising biocontrol agent against hazelnut pests [22]. In this study, when the LT_50_ and LT_90_ values of *M. anisopliae* isolates that were applied to *A. dispar*, *X. germanus*, and *X. saxesenii* adults were examined, it was understood that TR-55-019 was a more effective isolate than the others, and caused 100% death in all beetles by day 7. The results showed that all isolates of *M. anisopliae* are highly virulent against ambrosia beetles. Although the fungus is effective against some bark beetles [47,54,55], its effectiveness on ambrosia beetles was previously investigated in only a few studies [11,18,19]. Castrillo et al. [18] determined that *M. brunneum* F52, at 600 conidia/mm^2^, against female adults of *X. germanus* caused 61.7% mortality at 6 days. Similarly, the LT_50_ and LT_90_ values of *M. anisopliae* (TR-106) at 1 × 10^8^ spore mL^−1^ against female adults of *X. germanus* were 4.43 days and 6.01 days, respectively, and a 100% mortality rate occurred at the end of the day 8 [11].

Other EPFs isolated in the present study were *P. lilacinum* and *C. rosea*. *Purpureocillium lilacinum* was isolated from insects in tropical regions [56]. However, there is no existing study on the isolation of these fungi from bark and ambrosia beetles or their pathogenicity against the beetles. In this study, it was found that *P. lilacinum* isolates isolated caused 60% to 72% mortality in *A. dispar* and *X. germanus* adults within 9 days. These results showed that *P. lilacinum* was moderately effective on these ambrosia beetles. On the other hand, *C. rosea* is a fungus that is generally known to be mycoparasitic, but it has been reported as an entomopathogen in a few studies [57]. There is no current study showing that this fungus has been isolated from ambrosia beetles or that it has been tested on these beetles. In addition, the *C. rosea* isolates used in this study were found to have very low efficiency as entomopathogens.

In pathogenicity studies, it was determined that the activity of EPF on *A. dispar*, *X. germanus*, and *X. saxesenii* varied considerably between species and even between different isolates of the same species. According to Goettel et al. [58] and Sevim et al. [22], the virulence of EPF may differ between genera, within a genus, and even between species isolates, due to genetic diversity.

## 5. Conclusions

Applying biopesticides based on EPF in the biocontrol of insect pests is advantageous and environmentally friendly in many ways. Currently, many studies are being carried out to identify and characterize EPF obtained from different sources. In this study, we identified novel EPF isolates that have the potential for use in the myco-biocontrol of ambrosia beetles that cause significant damage in hazelnut orchards. These EPF isolates were successfully characterized with the use of molecular tools, including ITS sequencing and iPBS profiling. All of the isolates were tested for virulence against *A. dispar*, *X. germanus*, and *X. saxesenii*, and the pathogenicity varied among the isolates. The pathogenicity of the EPF isolates on these beetles was concentration- and time-dependent. There is a need for further studies, with more effective isolates, before the production of commercial products for use in field applications.

## Figures and Tables

**Table 1:** Information about isolated entomopathogenic fungi.

No.	Isolate Code	Species	Isolated Beetle	Collected Location/Province	GenBank Accession Nos
1	TR-55-034	*B. bassiana*	*X. germanus*	Ondokuz Mayıs/Samsun	MN588126
2	TR-55-006	*B. bassiana*	*A. dispar*	Terme/Samsun	MN588120
3	TR-52-002	*B. bassiana*	*A. dispar*	Fatsa/Ordu	MN588119
4	TR-52-003	*B. bassiana*	*A. dispar*	Ünye/Ordu	MN588125
5	TR-52-004	*B. bassiana*	*X. germanus*	Fatsa/Ordu	MN588122
6	TR-54-002	*B. bassiana*	*A. dispar*	Akyazı/Sakarya	MN588117
7	TR-54-004	*B. bassiana*	*A. dispar*	Akyazı/Sakarya	MN588121
8	TR-28-012	*B. bassiana*	*X. germanus*	Piraziz/Giresun	MN588118
9	TR-28-003	*B. bassiana*	*X. germanus*	Merkez/Giresun	MN588124
10	TR-28-004	*B. bassiana*	*X. saxesenii*	Merkez/Giresun	MN588127
11	TR-52-009	*B. bassiana*	*X. germanus*	Ünye/Ordu	MN588123
12	TR-55-001	*B. pseudobassiana*	*X. germanus*	Çarşamba/Samsun	MN588111
13	TR-55-003	*B. pseudobassiana*	*A. dispar*	Çarşamba/Samsun	MN588112
14	TR-55-004	*B. pseudobassiana*	*X. saxesenii*	Ondokuz Mayıs/Samsun	MN588113
15	TR-55-024	*B. pseudobassiana*	*X. germanus*	Ondokuz Mayıs/Samsun	MN588116
16	TR-55-030	*B. pseudobassiana*	*X. germanus*	Terme/ Samsun	MN588109
17	TR-52-001	*B. pseudobassiana*	*X. germanus*	Fatsa/ Ordu	MN588110
18	TR-28-001	*B. pseudobassiana*	*A. dispar*	Merkez/Giresun	MN588114
19	TR-28-002	*B. pseudobassiana*	*A. dispar*	Buluncak/Giresun	MN588115
20	TR-55-002	*C. fumosorosea*	*X. germanus*	Ondokuz Mayıs/ Samsun	MN588101
21	TR-55-015	*C. fumosorosea*	*A. dispar*	Çarşamba/Samsun	MN588099
22	TR-55-016	*C. fumosorosea*	*X. germanus*	Terme/ Samsun	MN588098
23	TR-55-018	*C. fumosorosea*	*X. saxesenii*	Terme/ Samsun	MN588103
24	TR-28-010	*C. fumosorosea*	*A. dispar*	Piraziz/ Giresun	MN588102
25	TR-54-007	*C. fumosorosea*	*A. dispar*	Hendek/ Sakarya	MN588100
26	TR-52-014	*C. farinosa*	*A. dispar*	Ünye/ Ordu	MN588141
27	TR-55-020	*A. lecanii*	*A. dispar*	Terme/ Samsun	MN588133
28	TR-55-033	*A. lecanii*	*X. saxesenii*	Çarşamba/ Samsun	MN588132
29	TR-54-001	*A. lecanii*	*A. dispar*	Akyazı/ Sakarya	MN588140
30	TR-81-001	*A. lecanii*	*A. dispar*	Gülyaka	MN588129
31	TR-81-002	*A. lecanii*	*A. dispar*	Cumayeri/Düzce	MN588130
32	TR-81-003	*A. lecanii*	*X. germanus*	Cumayeri/Düzce	MN588128
33	TR-81-004	*A. lecanii*	*A. dispar*	Gülyaka/Düzce	MN588131
34	TR-81-005	*A. lecanii*	*A. dispar*	Gülyaka/Düzce	MN588137
35	TR-54-003	*A. lecanii*	*A. dispar*	Hendek/Sakarya	MN588135
36	TR-54-008	*A. lecanii*	*X. germanus*	Hendek/Sakarya	MN588138
37	TR-52-006	*A. lecanii*	*X. germanus*	Merkez/Ordu	MN588139
38	TR-28-007	*A. lecanii*	*X. germanus*	Bulancak/Giresun	MN588136
39	TR-28-008	*A. lecanii*	*X. saxesenii*	Bulancak/Giresun	MN588134
40	TR-52-007	*P. lilacinum*	*X. germanus*	Ünye/Ordu	MN588106
41	TR-52-010	*P. lilacinum*	*A. dispar*	Gülyalı/Giresun	MN588105
42	TR-28-005	*P. lilacinum*	*A. dispar*	Keşap/Giresun	MN588104
43	TR-55-010	*C. rosea*	*X. germanus*	Ondokuz Mayıs/Samsun	MN588107
44	TR-28-006	*C. rosea*	*A. dispar*	Bulancak/Giresun	MN588108
45	TR-55-019	*M. anisopliae*	*X. germanus*	Ondokuz Mayıs/Samsun	MN588143
46	TR-54-005	*M. anisopliae*	*A. dispar*	Hendek/Sakarya	MN588144
47	TR-54-006	*M. anisopliae*	*X. germanus*	Hendek/Sakarya	MN588142

**Table 2:** Information on the PBS primers used to evaluate entomopathogenic fungal isolates.

Primer ID	Sequences (5′ to 3′)	Ta (°C)	GC (%)	TB	PB	PPB (%)	PIC	RP
2395	TCCCCAGCGGAGTCGCCA	62	72.2	26	25	96.15	0.20	6.30
2386	CTGATCAACCCA	50	50.0	23	22	95.65	0.25	7.79
2415	CATCGTAGGTGGGCGCCA	60	66.7	25	23	92.00	0.24	8.13
2242	GCCCCATGGTGGGCGCCA	62	77.8	19	18	94.74	0.24	5.64
2080	CAGACGGCGCCA	55	75.0	18	17	94.44	0.22	4.64
2221	ACCTAGCTCACGATGCCA	58	55.6	27	25	92.59	0.26	9.06
2381	GTCCATCTTCCA	50	50.0	18	17	94.44	0.21	4.77
2239	ACCTAGGCTCGGATGCCA	60	61.1	29	28	96.55	0.25	8.89
2219	GAACTTATGCCGATACCA	55	44.4	22	21	95.45	0.25	7.15
2390	GCAACAACCCCA	50	58.3	30	29	96.67	0.24	9.02
	Total			237	225			
	Average/primer			23.70	22.50	94.87	0.24	7.14

Ta (°C): optimal annealing temperature; GC (%): percentage of guanine-cytosine content; TB: total band; PB: polymorphic band; PPB (%): percentage of the polymorphic band; PIC: polymorphism information content; RP: resolving power.

**Table 3:** Nei’s genetic distance matrix of eight EPF species obtained from iPBS-PCR.

	*A. le*	*B. ba*	*B. ps*	*C. fa*	*C. fu*	*C. ro*	*M. an*
*Beauveria bassiana*	0.143						
*Beauveria pseudobassiana*	0.311	0.282					
*Cordyceps farinosa*	0.072	0.132	0.274				
*Cordyceps fumosorosea*	0.183	0.200	0.362	0.150			
*Clonotachys rosea*	0.193	0.212	0.361	0.162	0.223		
*Metarhizium anisopliae*	0.196	0.220	0.369	0.155	0.230	0.136	
*Purpureocillium lilacinum*	0.144	0.166	0.308	0.116	0.163	0.162	0.185

## Data Availability

All relevant data generated or analysed during this study are included in this manuscript.

## Supplementary Materials

The following supporting information can be downloaded at: https://www.mdpi.com/article/10.3390/insects13090824/s1, Table S1: Probit analysis data on mortality time (days) of *Anisandrus dispar* after application of 1 × 10^8^ spore mL^−1^ concentration of *Beauveria bassiana* isolates; Table S2: Probit analysis data on mortality time (days) of *Anisandrus dispar* after applications of 1 × 10^8^ spore mL^−1^ concentration of *Beauveria pseudobassiana* isolates; Table S3: Probit analysis data on mortality time (days) of *Anisandrus dispar* after applications of 1 × 10^8^ spore mL^−1^ concentration of *Metarhizium anisopliae* isolates; Table S4: Probit analysis data on mortality time (days) of *Anisandrus dispar* after applications of 1 × 10^8^ spore mL^−1^ concentration of *Cordyceps fumosorosea and C. farinosa* isolates; Table S5: Probit analysis data on mortality time (days) of *Anisandrus dispar* after applications of 1 × 10^8^ spore mL^−1^ concentration of *Akanthomyces lecanii* isolates; Table S6: Probit analysis data on mortality time (days) of *Anisandrus dispar* after applications of 1 × 10^8^ spore mL^−1^ concentration of *Purpureocillium lilacinum* isolates; Table S7: Probit analysis data on mortality time (days) of *Anisandrus dispar* after applications of 1 × 10^8^ spore mL^−1^ concentration of *Clonostachys rosea* isolates; Table S8: Probit analysis data on mortality time (days) of *Xylosandrus germanus* after application of 1 × 10^8^ spore mL^−1^ concentration of *Beauveria bassiana* isolates; Table S9: Probit analysis data on mortality time (days) of *Xylosandrus germanus* after applications of 1 × 10^8^ spore mL^−1^ concentration of *Beauveria pseudobassiana* isolates; Table S10: Probit analysis data on mortality time (days) of *Xylosandrus germanus* after applications of 1 × 10^8^ spore mL^−1^ concentration of *Metarhizium anisopliae* isolates; Table S11: Probit analysis data on mortality time (days) of *Xylosandrus germanus* after applications of 1 × 10^8^ spore mL^−1^ concentration of *Cordyceps fumosorosea and C. farinosa* isolates; Table S12: Probit analysis data on mortality time (days) of *Xylosandrus germanus* after applications of 1 × 10^8^ spore mL^−1^ concentration of *Akanthomyces lecanii* isolates; Table S13: Probit analysis data on mortality time (days) of *Xylosandrus germanus* after applications of 1 × 10^8^ spore mL^−1^ concentration of *Purpureocillium lilacinum* isolates; Table S14: Probit analysis data on mortality time (days) of *Xylosandrus germanus* after applications of 1 × 10^8^ spore mL^−1^ concentration of *Clonostachys rosea* isolates; Table S15: Probit analysis data on mortality time (days) of *Xyleborinus saxesenii* after applications of 1 × 10^8^ spore mL^−1^ concentration of entomopathogenic fungus isolates; Figure S1: Mortality rates of *Anisandrus dispar* treated with *Beauveria bassiana* isolates at 1 × 10^8^ spore mL^−1^ concentration (a,b); Figure S2: Mortality rates of *Anisandrus dispar* treated with *Beauveria pseudobassiana* isolates at 1 × 10^8^ spore mL^−1^ concentration (a,b); Figure S3: Mortality rates of *Anisandrus dispar* treated with *Metarhizium anisopliae* isolates at 1 × 10^8^ spore mL^−1^ concentration; Figure S4: Mortality rates of *Anisandrus dispar* treated with *Cordyceps fumosorosea and C. farinosa* isolates at 1 × 10^8^ spore mL^−1^ concentration; Figure S5: Mortality rates of *Anisandrus dispar* treated with *Akanthomyces lecanii* isolates at 1 × 10^8^ spore mL^−1^ concentration (a,b); Figure S6: Mortality rates of *Anisandrus dispar* treated with *Purpureocillium lilacinum* isolates at 1 × 10^8^ spore mL^−1^ concentration; Figure S7: Mortality rates of *Anisandrus dispar* treated with *Clonostachys rosea* isolates at 1 × 10^8^ spore mL^−1^ concentration; Figure S8: Mortality rates of *Xylosandrus germanus* treated with *Beauveria bassiana* isolates at 1 × 10^8^ spore mL^−1^ concentration (a,b); Figure S9: Mortality rates of *Xylosandrus germanus* treated with *Beauveria pseudobassiana* isolates at 1 × 10^8^ spore mL^−1^ concentration (a,b); Figure S10: Mortality rates of *Xylosandrus germanus* treated with *Metarhizium anisopliae* isolates at 1 × 10^8^ spore mL^−1^ concentration; Figure S11: Mortality rates of *Xylosandrus germanus* treated with *Cordyceps fumosorosea and C. farinosa* isolates at 1 × 10^8^ spore mL^−1^ concentration; Figure S12: Mortality rates of *Xylosandrus germanus* treated with *Akanthomyces lecanii* isolates at 1 × 10^8^ spore mL^−1^ concentration (a,b); Figure S13: Mortality rates of *Xylosandrus germanus* treated with *Purpureocillium lilacinum* isolates at 1 × 10^8^ spore mL^−1^ concentration; Figure S14: Mortality rates of *Xylosandrus germanus* treated with *Clonostachys rosea* isolates at 1 × 10^8^ spore mL^−1^ concentration; Figure S15: Mortality rates of *Xyleborinus saxesenii* treated with entomopathogenic fungus isolates at 1 × 10^8^ spore mL^−1^ concentration (a,b).
