# Supplementary material for: Molecular Characterization of Native Entomopathogenic Fungi from Ambrosia Beetles in Hazelnut Orchards of Turkey and Evaluation of Their In Vitro Efficacy
Source: Insects. 2022 Sep 11;13(9):824. doi: 10.3390/insects13090824 (PMC9502873; doi:10.3390/insects13090824)
Supplement: Supplementary file 1 [file insects-13-00824-s001.zip › insects-1889532-supplementary.pdf]

Table S1. Probit analysis data on mortality time (days) of *Anisandrus dispar* after application of 1 × 10^8 spore mL^{-1} concentration of *Beauveria bassiana* isolates

Isolates	LT_{50} (95% CI)	LT_{90} (95% CI)	Slope ± SE	Regression	X^2	Df	Heterogeneity
TR-55-034	4.84(4.46-5.21)b*	7.24(6.60-8.27)ab*	7.33±0.89	y=-5.02+7.33x	13.31	43	0.31
TR-55-006	4.12(3.78-4.44)a	5.96(5.45-6.76)a	7.97±0.98	y=-4.90+7.97x	40.61	43	0.94
TR-52-002	4.95(4.58-5.30)bc	7.14(6.54-8.08)ab	8.06±0.99	y=-5.59+8.06x	17.06	43	0.40
TR-52-003	5.09(4.72-5.45)bc	7.32(6.71-8.31)ab	8.10±0.99	y=-5.71+8.10x	21.64	43	0.50
TR-52-004	5.11(4.75-5.46)bc	7.18(6.62-8.07)ab	8.68±1.07	y=-6.15+8.68x	18.41	43	0.41
TR-54-002	5.69(5.29-6.09)c	8.22(7.51-9.42)bc	8.01±1.03	y=-6.04+8.01x	14.89	43	0.35
TR-54-004	5.18(4.82-5.53)bc	7.27(6.70-8.18)ab	8.70±1.08	y=-6.21+8.70x	17.74	43	0.41
TR-28-012	5.34(4.98-5.71)bc	7.58(6.96-8.58)b	8.45±1.05	y=-6.14+8.45x	23.70	43	0.55
TR-28-003	9.70(8.64-12.77)e	14.87(11.73-28.48)cd	6.91±1.69	y=-6.82+6.91x	8.39	43	0.20
TR-28-004	11.77(9.52-21.82)e	22.67(14.87-86.04)d	4.51±1.22	y=-4.82+4.51x	20.94	43	0.49
TR-52-009	6.67(6.19-7.24)d	10.26(9.07-12.60)c	6.85±0.99	y=-5.64+6.85x	12.15	43	0.28

*Within columns, means followed by the same lower-case letters do not differ significantly at $p \leq 0.05$.

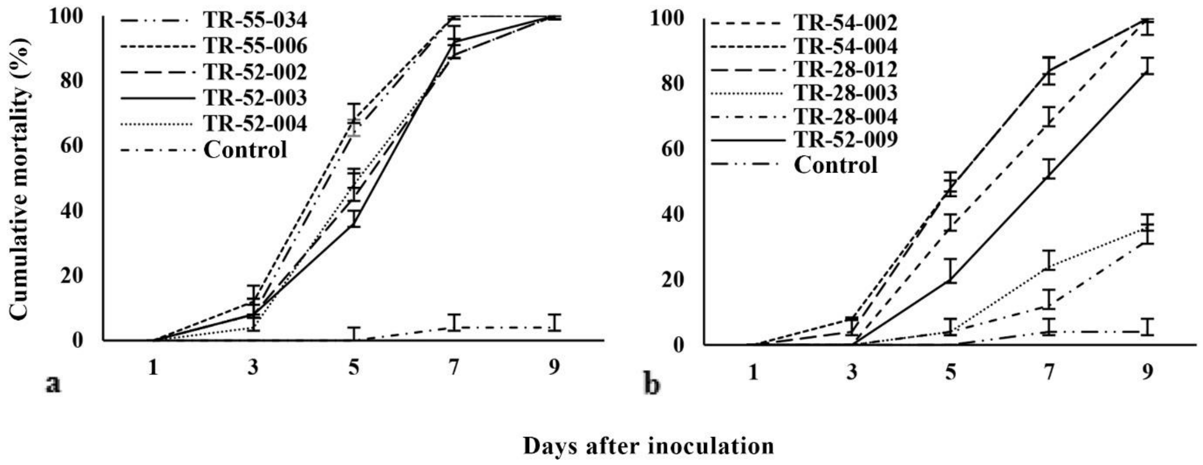

Figure S1. Mortality rates of *Anisandrus dispar* treated with *Beauveria bassiana* isolates at 1 × 10^8 spore mL^{-1} concentration (a, b).

Table S2. Probit analysis data on mortality time (days) of *Anisandrus dispar* after applications of 1 × 10^8 spore mL^{-1} concentration of *Beauveria pseudobassiana* isolates

Isolates	LT_{50} (95% CI)	LT_{90} (95% CI)	Slope ± SE	Regression	X^2	Df	Heterogeneity
TR-55-001	4.91(4.51-5.30)ab*	7.54(6.84-8.70)ab*	6.87±0.85	y=-4.75+6.87x	14.41	43	0.34
TR-55-003	7.54(6.71-8.93)c	15.24(11.89-24.51)c	4.20±0.71	y=-3.69+4.20x	13.71	43	0.32
TR-55-004	5.63(5.20-6.07)b	8.71(7.83-10.22)bc	6.76±0.85	y=-5.08+6.76x	18.41	43	0.43
TR-55-024	5.17(4.75-5.57)ab	7.91(7.17-9.12)b	6.93±0.85	y=-4.94+6.93x	17.61	43	0.41
TR-55-030	4.91(4.49-5.31)ab	7.76(7.00-9.02)ab	6.43±0.79	y=-4.44+6.43x	16.51	43	0.38
TR-52-001	4.56(4.24-4.88)a	6.28(5.79-7.05)a	9.25±1.18	y=-6.09+9.25x	13.02	43	0.30
TR-28-001	6.71(6.12-7.49)c	11.86(10.00-15.97)c	5.19±0.79	y=-4.29+5.19x	8.09	43	0.19
TR-28-002	-**	-**	-	-	-	-	-

*Within columns, means followed by the same lower-case letters do not differ significantly at $p \leq 0.05$.
**Because of the extremely low mortality rates, LT_{50} and LT_{90} values were not be calculated.

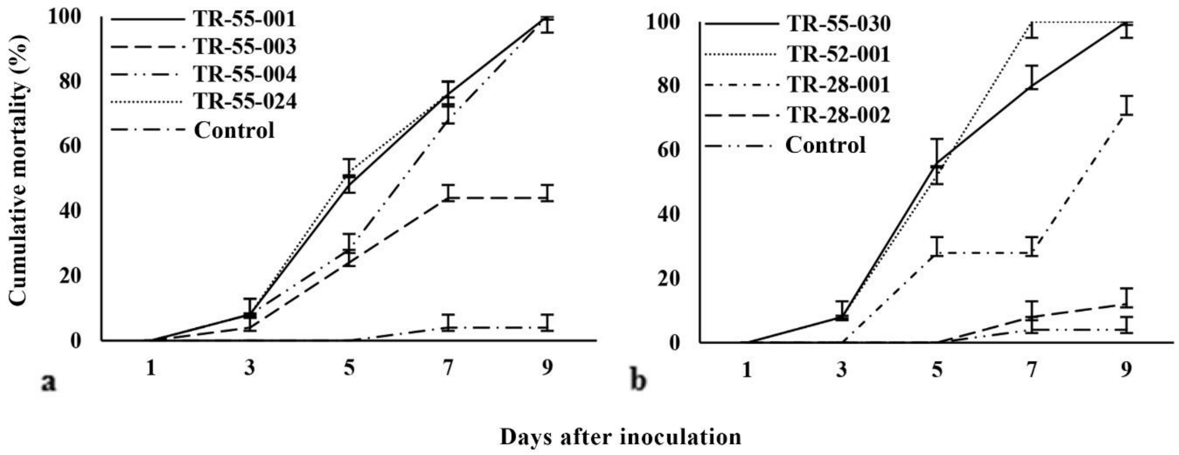

Figure S2. Mortality rates of *Anisandrus dispar* treated with *Beauveria pseudobassiana* isolates at 1 × 10^8 spore mL^{-1} concentration (a, b).

Table S3. Probit analysis data on mortality time (days) of *Anisandrus dispar* after applications of 1 × 10^8 spore mL^{-1} concentration of *Metarhizium anisopliae* isolates

Isolates	LT_{50} (95% CI)	LT_{90} (95% CI)	Slope ± SE	Regression	X^2	Df	Heterogeneity
TR-55-019	3.63(3.33-3.92)a*	5.02(4.60-5.71)a*	9.11±1.24	y=-5.11+9.11x	25.94	43	0.60
TR-54-005	4.28(3.96-4.58)b	5.88(5.42-6.61)ab	9.28±1.23	y=-5.85+9.28x	11.81	43	0.28
TR-54-006	4.79(4.41-5.15)b	7.04(6.44-8.01)b	7.65±0.94	y=-5.20+7.65x	13.90	43	0.32

*Within columns, means followed by the same lower-case letters do not differ significantly at $p \leq 0.05$.

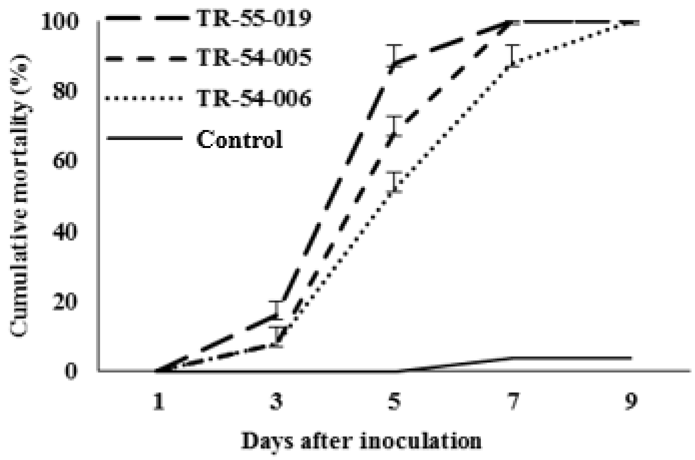

Figure S3. Mortality rates of *Anisandrus dispar* treated with *Metarhizium anisopliae* isolates at 1 × 10^8 spore mL^{-1} concentration

Table S4. Probit analysis data on mortality time (days) of *Anisandrus dispar* after applications of 1 × 10^8 spore mL^{-1} concentration of *Cordyceps fumosorosea and C. farinosa* isolates

Isolates	LT_{50} (95% CI)	LT_{90} (95% CI)	Slope ± SE	Regression	X^2	Df	Heterogeneity
TR-55-002	5.19(4.77-5.62)a*	8.21(7.37-9.61)a*	6.45±0.80	y=-4.62+6.45x	19.82	43	0.46
TR-55-015	8.86(7.68-11.42)b	18.38(13.47-35.79)b	4.04±0.78	y=-3.83+4.04x	16.97	43	0.40
TR-55-016	8.70(7.78-10.61)b	15.08(11.89-25.31)b	5.36±1.06	y=-5.03+5.36x	9.18	43	0.21
TR-55-018	5.12(4.71-5.52)a	7.84(7.10-9.05)a	6.94±0.86	y=-4.92+6.94x	13.60	43	0.32
TR-28-010	9.98(8.70-13.83)b	16.71(12.57-36.04)b	5.73±1.38	y=-5.72+5.73x	10.37	43	0.24
TR-54-007	9.23(8.14-11.84)b	16.26(12.44-30.41)b	5.21±1.10	y=-5.03+5.21x	10.41	43	0.24
TR-52-014	10.55(9.17-16.73)b	15.78(11.98-43.13)b	7.32± 2.17	y=-7.49+7.32x	11.56	43	0.27

*Within columns, means followed by the same lower-case letters do not differ significantly at $p \leq 0.05$.

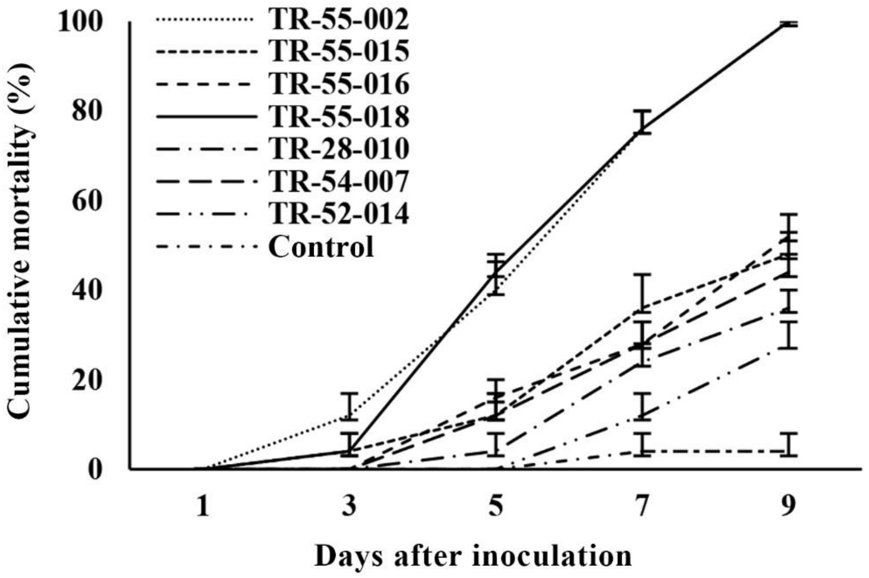

Figure S4. Mortality rates of *Anisandrus dispar* treated with *Cordyceps fumosorosea and C. farinosa* isolates at 1 × 10^8 spore mL^{-1} concentration

Table S5. Probit analysis data on mortality time (days) of *Anisandrus dispar* after applications of 1 × 10^8 spore mL^{-1} concentration of *Akanthomyces lecanii* isolates

Isolates	LT_{50} (95% CI)	LT_{90} (95% CI)	Slope ± SE	Regression	X^2	Df	Heterogeneity
TR-55-020	10.43(8.79-15.58)c*	20.12(14.08-51.41)b*	4.50±1.05	y=-4.58+4.50x	14.14	43	0.33
TR-55-033	9.57(8.54-12.41)c	14.88(11.75-27.85)b	6.68±1.59	y=-6.55+6.68x	10.89	43	0.25
TR-54-001	9.92(8.41-14.04)c	20.13(14.17-47.11)b	4.17±0.91	y=-4.15+4.17x	12.70	43	0.30
TR-81-001	8.82(7.73-11.19)c	17.15(12.88-31.92)b	4.44±0.87	y=-4.20+4.44x	9.93	43	0.23
TR-81-002	11.31(9.24-19.25)c	22.52(14.93-74.26)b	4.28±1.09	y=-4.51+4.28x	18.31	43	0.43
TR-81-003	-**	-**	-	-	-	-	-
TR-81-004	4.72(4.34-5.09)a	7.10(6.46-8.11)a	7.25±0.88	y=-4.88+7.25x	13.58	43	0.32
TR-81-005	9.84(8.67-13.25)c	15.77(12.16-31.74)b	6.25±1.51	y=-6.21+6.25x	13.25	43	0.31
TR-54-003	4.83(4.46-5.19)a	7.05(6.46-7.99)a	7.81±0.96	y=-5.34+7.81x	12.91	43	0.30
TR-54-008	9.72(8.68-12.80)c	14.64(11.62-28.04)b	7.20±1.79	y=-7.10+7.20x	10.15	43	0.24
TR-52-006	6.31(5.70-7.07)b	11.97(11.97-16.37)b	4.60±0.69	y=-3.68+4.60x	12.80	43	0.30
TR-28-007	5.17(4.80-5.54)a	7.43(6.82-8.43)a	8.15±1.03	y=-5.81+8.15x	13.65	43	0.32
TR-28-008	4.91(4.53-5.27)a	7.18(6.56-8.15)a	7.77±0.95	y=-5.36+7.77x	13.45	43	0.31

*Within columns, means followed by the same lower-case letters do not differ significantly at $p \leq 0.05$.
**Because of the extremely low mortality rates, LT_{50} and LT_{90} values were not be calculated.

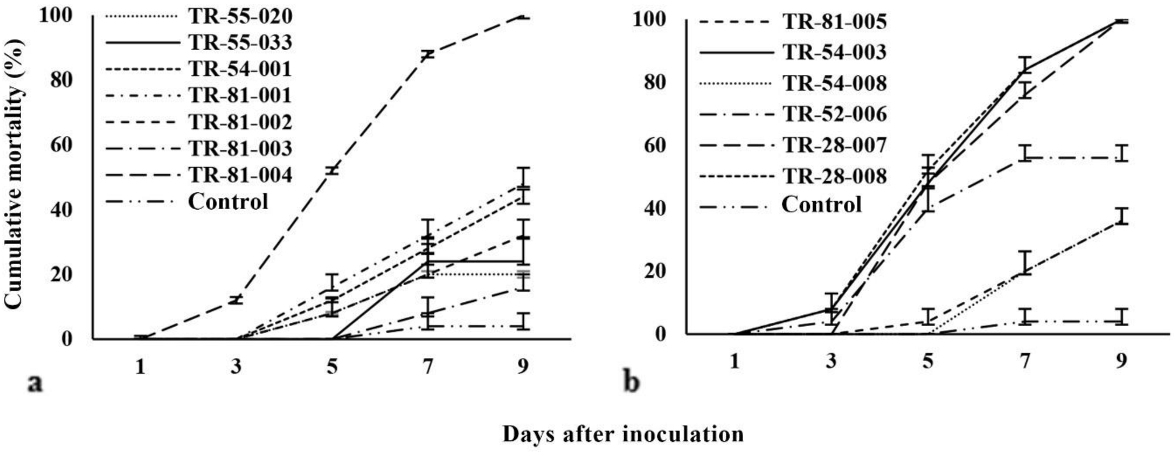

Figure S5. Mortality rates of *Anisandrus dispar* treated with *Akanthomyces lecanii* isolates at 1 × 10^8 spore mL^{-1} concentration (a, b)

Table S6. Probit analysis data on mortality time (days) of *Anisandrus dispar* after applications of 1 × 10^8 spore mL^{-1} concentration of *Purpureocillium lilacinum* isolates

Isolates	LT_{50} (95% CI)	LT_{90} (95% CI)	Slope ± SE	Regression	X^2	Df	Heterogeneity
TR-52-007	6.80(6.29-7.42)a*	10.69(9.36-13.38)a*	6.52±0.96	y=-5.42+6.52x	11.07	43	0.26
TR-52-010	7.67(7.05-8.62)a	12.32(10.42-16.87)a	6.23±1.05	y=-5.51+6.23x	11.13	43	0.26
TR-28-005	6.68(6.13-7.37)a	11.21(9.63-14.54)a	5.70±0.85	y=-4.69+5.69x	10.42	43	0.24

*Within columns, means followed by the same lower-case letters do not differ significantly at $p \leq 0.05$.

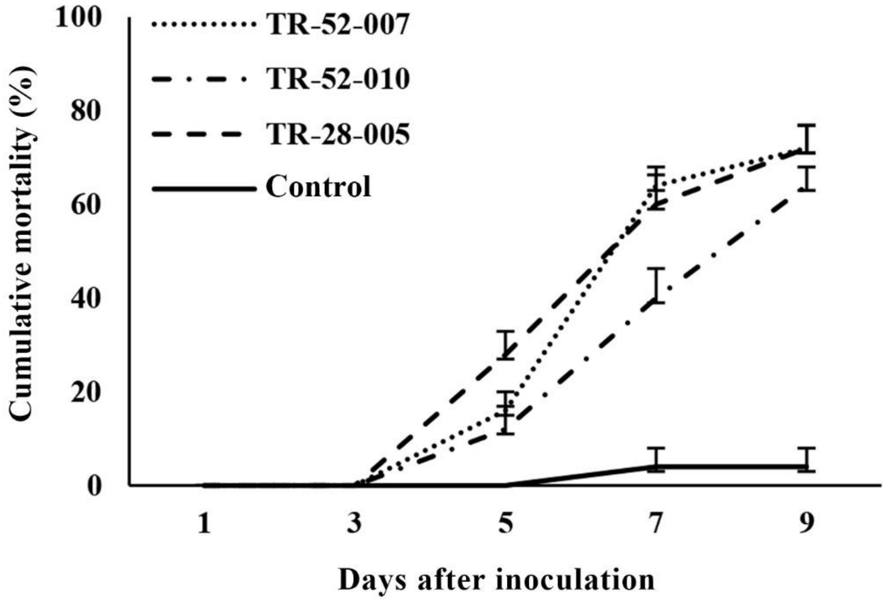

Figure S6. Mortality rates of *Anisandrus dispar* treated with *Purpureocillium lilacinum* isolates at 1 × 10^8 spore mL^{-1} concentration

Table S7. Probit analysis data on mortality time (days) of *Anisandrus dispar* after applications of 1 × 10^8 spore mL^{-1} concentration of *Clonostachys rosea* isolates

Isolates	LT_{50} (95% CI)	LT_{90} (95% CI)	Slope ± SE	Regression	X^2	Df	Heterogeneity
TR-55-010	11.66(9.58-24.66)a*	19.43(13.32-92.87)a*	5.77±1.82	y=-6.15+5.77x	11.14	43	0.26
TR-28-006	-**	-**	-	-	-	-	-

*Within columns, means followed by the same lower-case letters do not differ significantly at $p \leq 0.05$.
** Because of the extremely low mortality rates, LT_{50} and LT_{90} values were not be calculated.

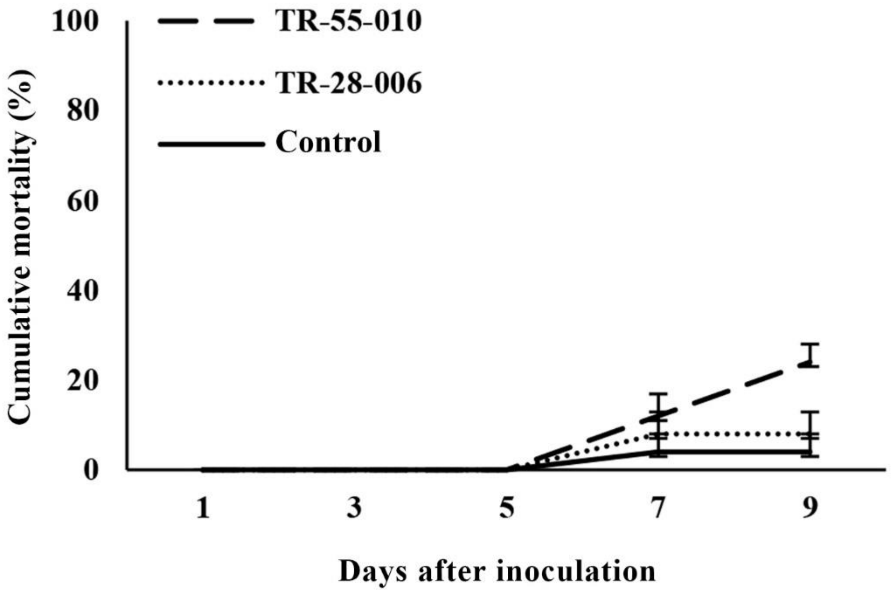

Figure S7. Mortality rates of *Anisandrus dispar* treated with *Clonostachys rosea* isolates at 1 × 10^8 spore mL^{-1} concentration

Table S8. Probit analysis data on mortality time (days) of *Xylosandrus germanus* after application of 1 × 10^8 spore mL^{-1} concentration of *Beauveria bassiana* isolates

Isolates	LT_{50} (95% CI)	LT_{90} (95% CI)	Slope ± SE	Regression	X^2	Df	Heterogeneity
TR-55-034	4.40(4.09-4.70)ab*	5.90(5.46-6.61)a*	10.05±1.34	y=-6.46+10.05x	12.13	43	0.28
TR-55-006	3.97(3.65-4.28)a	5.67(5.17-6.42)a	8.35±1.05	y=-4.99+8.34x	41.37	43	0.96
TR-52-002	4.96(4.61-5.29)b	6.86(6.33-7.68)ab	9.12±1.15	y=-6.34+9.12x	17.55	43	0.41
TR-52-003	5.45(5.08-5.81)bc	7.58(6.99-8.51)b	8.98±1.14	y=-6.61+8.97x	15.23	43	0.35
TR-52-004	4.89(4.56-5.19)b	6.42(5.98-7.11)ab	10.84±1.46	y=-7.46+10.84x	17.04	43	0.40
TR-54-002	5.18(4.83-5.53)bc	7.19(6.64-8.07)b	9.02±1.12	y=-6.44+9.02x	14.89	43	0.48
TR-54-004	5.57(5.12-6.03)bc	8.86(7.91-10.54)bc	6.34±0.81	y=-4.27+6.34x	20.96	43	0.49
TR-28-012	5.78(5.38-6.19)c	8.40(7.66-9.65)b	7.91±1.00	y=-6.02+7.91x	25.93	43	0.29
TR-28-003	9.75(8.66-12.96)e	15.04(11.80-29.55)c	6.80±1.68	y=-6.72+6.80x	9.54	43	0.22
TR-28-004	11.10(9.14-18.33)e	22.04(14.77-68.61)c	4.30±1.07	y=-4.50+4.30x	16.42	43	0.38
TR-52-009	7.62(7.04-8.46)d	11.78(10.12-15.65)c	6.78±1.14	y=-5.97+6.78x	10.51	43	0.24

*Within columns, means followed by the same lower-case letters do not differ significantly at $p \leq 0.05$.

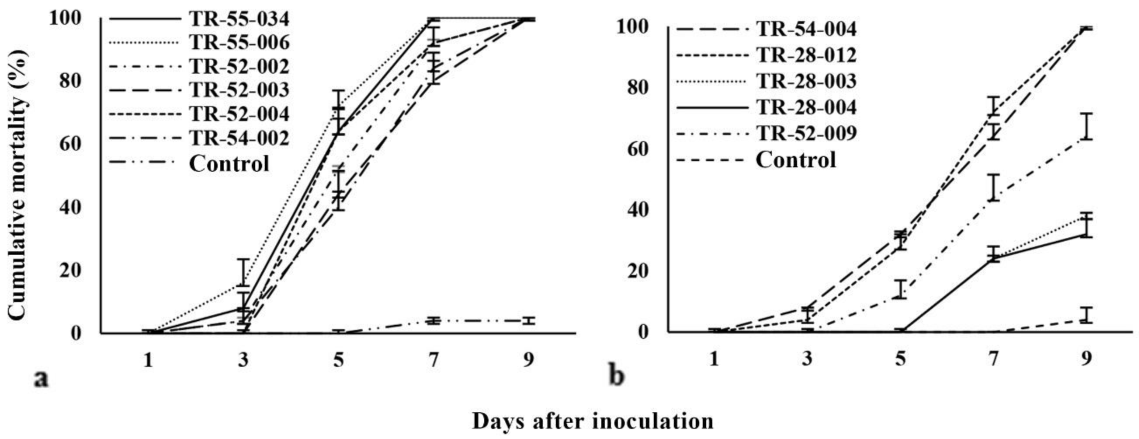

Figure S8. Mortality rates of *Xylosandrus germanus* treated with *Beauveria bassiana* isolates at 1 × 10^8 spore mL^{-1} concentration (a, b)

Table S9. Probit analysis data on mortality time (days) of *Xylosandrus germanus* after applications of 1 × 10^8 spore mL^{-1} concentration of *Beauveria pseudobassiana* isolates

Isolates	LT_{50} (95% CI)	LT_{90} (95% CI)	Slope ± SE	Regression	X^2	Df	Heterogeneity
TR-55-001	5.16(4.72-5.62)a*	8.50(7.57-10.08)a*	5.92±0.72	y=-4.21+5.92x	36.73	43	0.85
TR-55-003	5.88(5.52-6.25)ab	8.02(7.41-9.01)a	9.54±1.24	y=-7.34+9.54x	13.73	43	0.32
TR-55-004	6.03(5.59-6.49)ab	9.10(8.18-10.72)ab	7.16±0.94	y=-5.58+7.16x	26.39	43	0.61
TR-55-024	7.28(6.81-7.89)b	10.49(9.34-12.86)b	8.09±1.26	y=-6.97+8.08x	6.73	43	0.16
TR-55-030	5.40(4.99-5.78)a	7.86(7.19-8.95)a	7.84±0.10	y=-5.74+7.84x	12.78	43	0.30
TR-52-001	5.35(4.93-5.75)a	8.07(7.32-9.29)a	7.18±0.90	y=-5.22+7.18x	15.55	43	0.36
TR-28-001	6.72(6.06-7.62)b	12.91(10.57-18.37)b	4.52±0.70	y=-3.73+4.51x	13.78	43	0.32
TR-28-002	-**	-**	-	-	-	-	-

*Within columns, means followed by the same lower-case letters do not differ significantly at $p \leq 0.05$.
**Because of the extremely low mortality rates, LT_{50} and LT_{90} values were not be calculated.

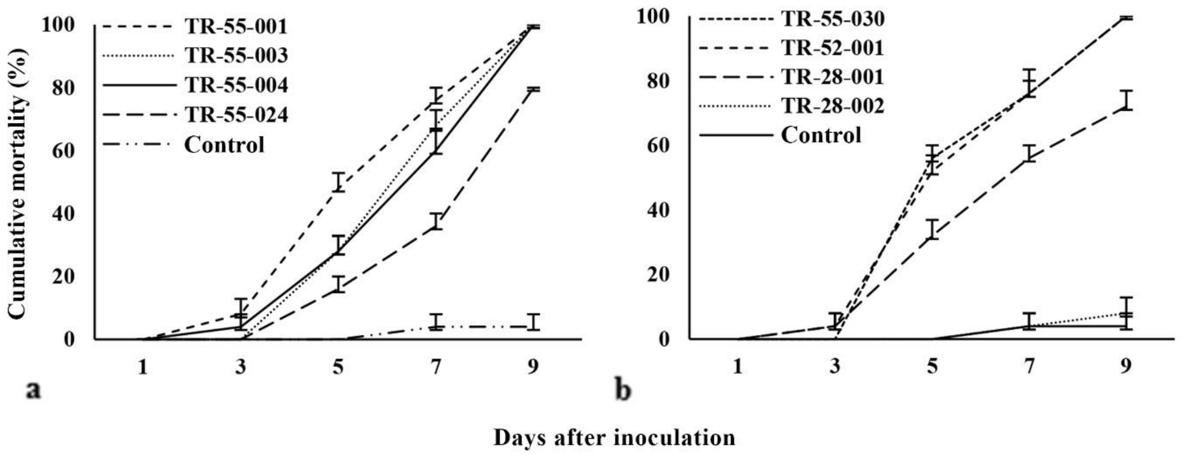

Figure S9. Mortality rates of *Xylosandrus germanus* treated with *Beauveria pseudobassiana* isolates at 1 × 10^8 spore mL^{-1} concentration (a, b)

Table S10. Probit analysis data on mortality time (days) of *Xylosandrus germanus* after applications of 1 × 10^8 spore mL^{-1} concentration of *Metarhizium anisopliae* isolates

Isolates	LT_{50} (95% CI)	LT_{90} (95% CI)	Slope ± SE	Regression	X^2	Df	Heterogeneity
TR-55-019	4.01(3.71-4.29)a*	5.40(4.97-6.10)a*	9.90±1.38	y=-5.96+9.89x	6.73	43	0.16
TR-54-005	4.47(4.10-4.84)ab	6.81(6.19-7.80)b	7.02±0.85	y=-4.56+7.02x	18.77	43	0.44
TR-54-006	5.19(4.77-5.61)b	8.08(7.29-9.41)b	6.66±0.83	y=-4.76+6.66x	18.39	43	0.43

*Within columns, means followed by the same lower-case letters do not differ significantly at $p \leq 0.05$.

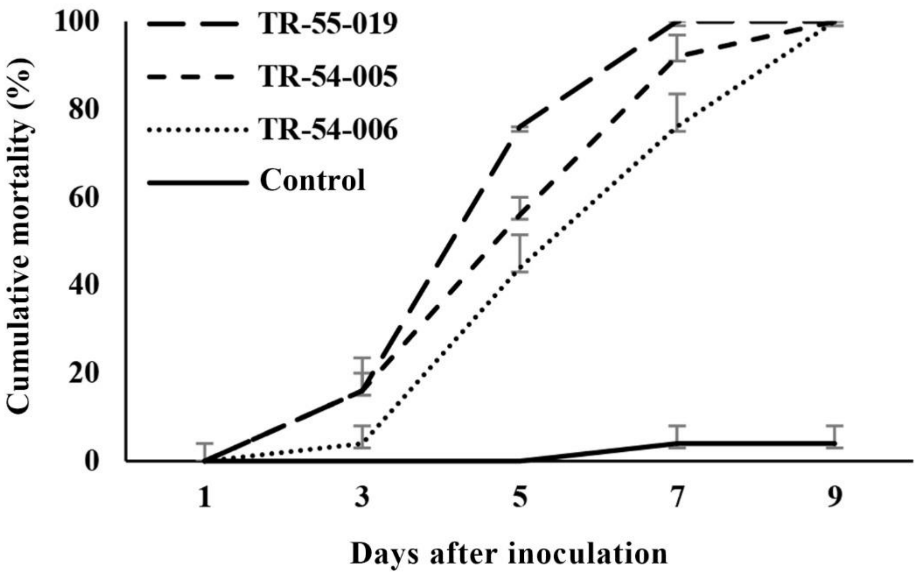

Figure S10. Mortality rates of *Xylosandrus germanus* treated with *Metarhizium anisopliae* isolates at 1 × 10^8 spore mL^{-1} concentration

Table S11. Probit analysis data on mortality time (days) of *Xylosandrus germanus* after applications of 1 × 10^8 spore mL^{-1} concentration of *Cordyceps fumosorosea and C. farinosa* isolates

Isolates	LT_{50} (95% CI)	LT_{90} (95% CI)	Slope ± SE	Regression	X^2	Df	Heterogeneity
TR-55-002	5.36(4.93-5.78)a*	8.33(7.50-9.73)a*	6.68±0.85	y=-4.87+6.68x	14.43	43	0.34
TR-55-015	6.53(6.03-7.13)b	10.44(9.15-13.01)ab	6.28±0.90	y=-5.12+6.28x	19.77	43	0.46
TR-55-016	9.53(8.35-12.50)c	16.73(16.73-32.34)b	5.24±1.13	y=-5.13+5.24x	21.68	43	0.50
TR-55-018	5.07(4.60-5.54)a	8.73(7.71-10.53)a	5.43±0.70	y=-3.83+5.43x	14.87	43	0.35
TR-28-010	10.61(9.14-16.73)c	16.62(12.38-45.31)b	6.57±1.86	y=-6.73+6.57x	10.41	43	0.24
TR-54-007	11.44(9.36-20.41)c	21.65(14.46-77.41)b	4.63±1.25	y=-4.89+4.63x	14.14	43	0.33
TR-52-014	11.15(9.41-20.51)c	17.59(12.67-63.76)b	6.47±1.99	y=-6.77+6.47x	12.65	43	0.29

*Within columns, means followed by the same lower-case letters do not differ significantly at $p \leq 0.05$.

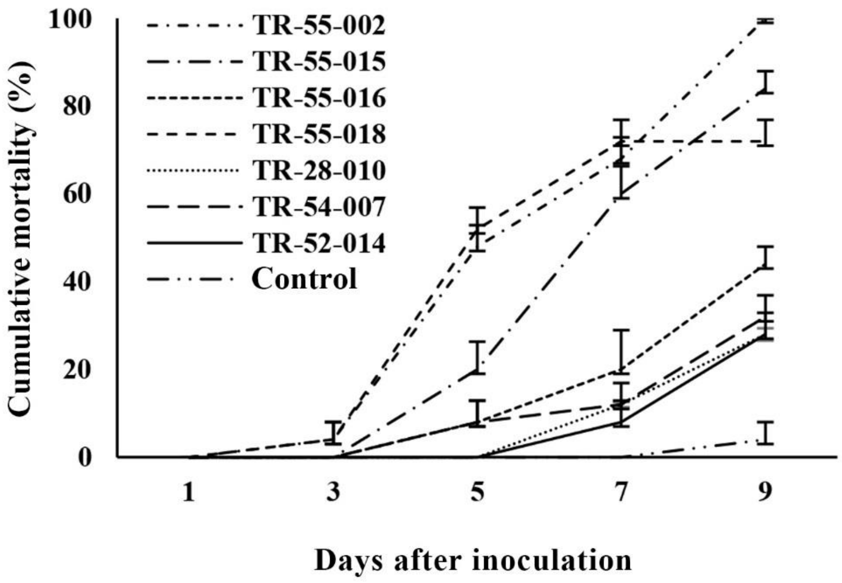

Figure S11. Mortality rates of *Xylosandrus germanus* treated with *Cordyceps fumosorosea and C. farinosa* isolates at 1 × 10^8 spore mL^{-1} concentration

Table S12. Probit analysis data on mortality time (days) of *Xylosandrus germanus* after applications of 1 × 10^8 spore mL^{-1} concentration of *Akanthomyces lecanii* isolates

Isolates	LT_{50} (95% CI)	LT_{90} (95% CI)	Slope ± SE	Regression	X^2	Df	Heterogeneity
TR-55-020	-**	-**	-	-	-	-	-
TR-55-033	9.58(8.31-12.84)d*	17.75(13.13-36.51)c*	4.78±1.03	y=-4.69+4.78x	11.34	43	0.39
TR-54-001	8.58(7.76-10.19)cd	14.08(11.43-21.90)c	5.96±1.14	y=-5.56+5.96x	16.76	43	0.42
TR-81-001	11.96(9.33-22.47)d	30.02(17.79-128.76)c	3.21±0.79	y=-3.45+3.21x	18.03	43	0.32
TR-81-002	9.86(8.51-13.54)d	18.01(13.23-38.58)c	4.90±1.09	y=-4.86+4.90x	13.59	43	0.54
TR-81-003	-**	-**	-	-	-	-	-
TR-81-004	4.43(4.11-4.74)a	6.06(5.59-6.79)a	9.43±1.23	y=-6.09+9.43x	10.59	43	0.55
TR-81-005	14.22(10.33-38.24)d	37.70(19.83-316.12)c	3.03±0.84	y=-3.49+3.02x	23.51	43	0.32
TR-54-003	4.62(4.26-4.96)a	6.68(6.13-7.56)ab	7.99±0.99	y=-5.30+7.99x	13.55	43	0.60
TR-54-008	10.11(8.83-14.20)d	16.30(12.37-35.54)c	6.18±1.54	y=-6.21+6.18x	12.31	43	0.29
TR-52-006	7.60(6.89-8.75)c	13.55(11.05-19.97)c	5.11±0.87	y=-4.50+5.11x	12.24	43	0.37
TR-28-007	5.56(5.17-5.96)b	8.10(7.40- 9.28)b	7.84±1.00	y=-5.84+7.84x	15.84	43	0.34
TR-28-008	6.33(5.82-6.92)bc	10.40(9.08-12.98)bc	5.95±0.83	y=-4.76+5.94x	14.64	43	0.26

*Within columns, means followed by the same lower-case letters do not differ significantly at $p \leq 0.05$.
**Because of the extremely low mortality rates, LT_{50} and LT_{90} values were not be calculated.

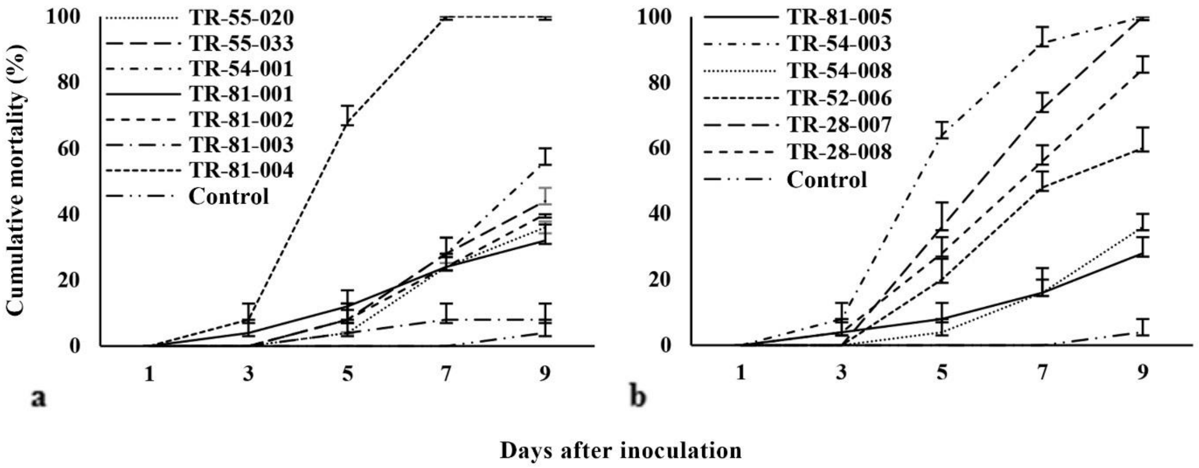

Figure S12. Mortality rates of *Xylosandrus germanus* treated with *Akanthomyces lecanii* isolates at 1 × 10^8 spore mL^{-1} concentration (a, b)

Table S13. Probit analysis data on mortality time (days) of *Xylosandrus germanus* after applications of 1 × 10^8 spore mL^{-1} concentration of *Purpureocillium lilacinum* isolates

Isolates	LT_{50} (95% CI)	LT_{90} (95% CI)	Slope ± SE	Regression	X^2	Df	Heterogeneity
TR-52-007	7.29(6.63-8.26)a*	12.81(10.63-17.95)a*	5.24±0.83	y=-4.52+5.23x	19.70	43	0.46
TR-52-010	7.72(7.00-8.90)a	13.57(11.08-20.05)a	5.22±0.90	y=-4.63+5.22x	10.38	43	0.24
TR-28-005	7.19(6.55-8.12)a	12.63(10.51-17.66)a	5.24±0.84	y=-4.49+5.24x	10.02	43	0.23

*Within columns, means followed by the same lower-case letters do not differ significantly at $p \leq 0.05$.

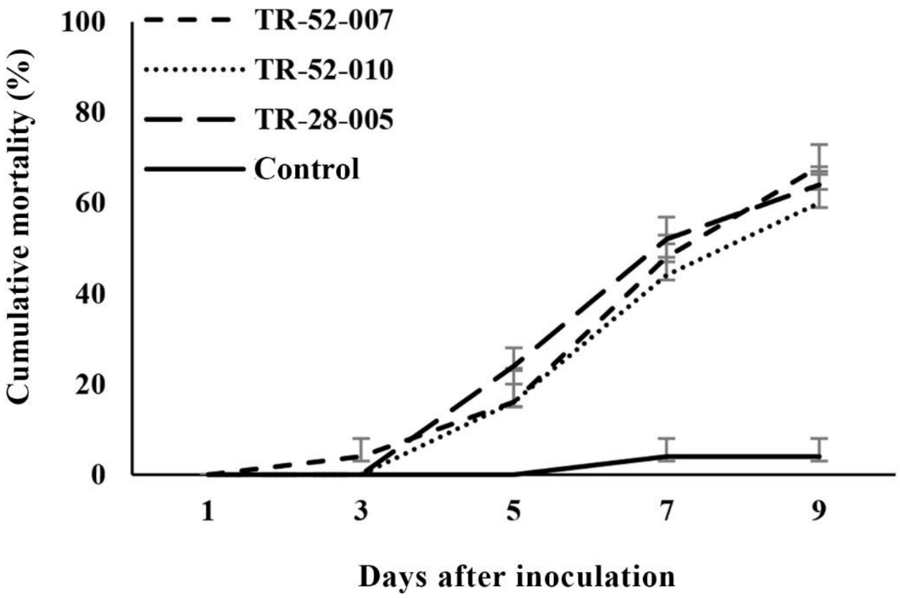

Figure S13. Mortality rates of *Xylosandrus germanus* treated with *Purpureocillium lilacinum* isolates at 1 × 10^8 spore mL^{-1} concentration

Table S14. Probit analysis data on mortality time (days) of *Xylosandrus germanus* after applications of 1 × 10^8 spore mL^{-1} concentration of *Clonostachys rosea* isolates

Isolates	LT_{50} (95% CI)	LT_{90} (95% CI)	Slope ± SE	Regression	X^2	Df	Heterogeneity
TR-55-010	-**	-**	-	-	-	-	-
TR-28-006	-**	-**	-	-	-	-	-

*Within columns, means followed by the same lower-case letters do not differ significantly at $p \leq 0.05$.
** Because of the extremely low mortality rates, LT_{50} and LT_{90} values were not be calculated..

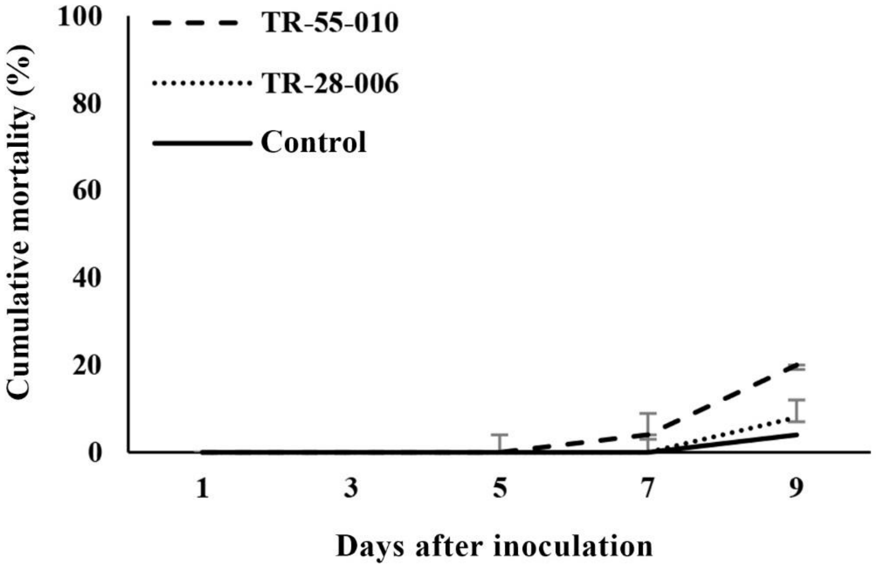

Figure S14. Mortality rates of *Xylosandrus germanus* treated with *Clonostachys rosea* isolates at 1 × 10^8 spore mL^{-1} concentration

Table S15. Probit analysis data on mortality time (days) of *Xyleborinus saxesenii* after applications of 1 × 10^8 spore mL^{-1} concentration of entomopathogenic fungus isolates

Isolates	LT_{50} (95% CI)	LT_{90} (95% CI)	Slope ± SE	Regression	X^2	Df	Heterogeneity
TR-55-034	5.08(4.74-5.40)bc*	6.80(6.31-7.57)a*	10.13±1.32	y=-7.14+10.13x	19.21	43	0.45
TR-55-006	3.71(3.38-4.03)a	5.57(5.06-6.37)a	7.26±0.88	y=-4.13+7.26x	21.29	43	0.50
TR-52-001	4.90(4.55-4.80)b	6.01(5.57-6.71)a	8.49±1.06	y=-5.86+8.49x	13.70	43	0.32
TR-55-004	5.79(5.38-6.24)c	8.74(7.89-10.22)b	7.18±0.92	y=-5.47+7.18x	21.14	43	0.49
TR-55-024	5.89(5.48-6.30)c	8.53(7.78-9.78)b	7.98±1.04	y=-6.14+7.98x	15.04	43	0.35
TR-55-016	5.53(4.92-6.23)c	11.72(9.64-16.21)bc	3.92±0.55	y=-2.91+3.92x	8.83	43	0.21
TR-55-002	5.32(4.82-5.84)c	9.50(8.26-11.81)b	5.09±0.67	y=-3.69+5.09x	12.69	43	0.30
TR-81-003	-**	-**	-	-	-	-	-
TR-81-002	9.13(7.56-12.90)d	25.26(16.43-64.39)c	2.90±0.57	y=-2.78+2.90x	18.82	43	0.44
TR-81-004	4.57(4.19-4.94)bc	6.93(6.31-7.93)ab	7.08±0.86	y=-4.67+7.08x	13.12	43	0.31
TR-55-033	9.59(8.37-12.72)d	17.07(12.83-33.85)c	5.12±1.11	y=-5.03+5.12x	16.32	43	0.38
TR-55-019	4.28(3.93-4.60)ab	6.12(5.61-6.92)a	8.24±1.04	y=-5.20+8.23x	11.41	43	0.27
TR-54-005	4.50(4.18-4.80)b	6.01(5.57-6.71)a	10.17±1.36	y=-6.36+10.17x	10.28	43	0.24

*Within columns, means followed by the same lower-case letters do not differ significantly at $p \leq 0.05$.
** Because of the extremely low mortality rates, LT_{50} and LT_{90} values were not be calculated..

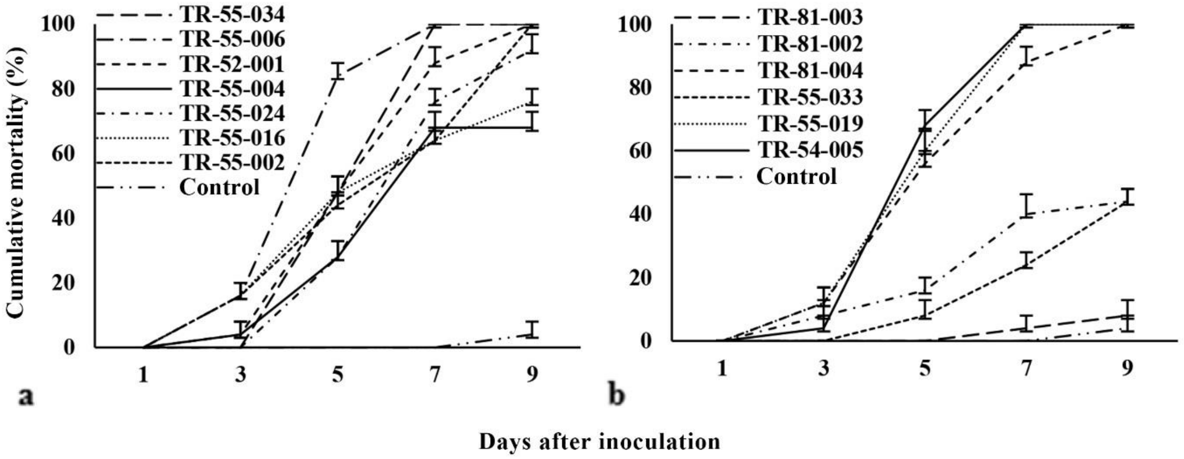

Figure S15. Mortality rates of *Xyleborinus saxesenii* treated with entomopathogenic fungus isolates at 1 × 10^8 spore mL^{-1} concentration (a, b)
